# Combining gamma neuromodulation and robotic rehabilitation after a stroke restores parvalbumin interneuron dynamics and improves motor recovery in mice

**DOI:** 10.1371/journal.pbio.3002806

**Published:** 2025-10-14

**Authors:** Livia Vignozzi, Francesca Macchi, Elena Montagni, Maria Pasquini, Alessandra Martello, Antea Minetti, Éléa Coulomb, Anna Letizia Allegra Mascaro, Silvestro Micera, Matteo Caleo, Cristina Spalletti

**Affiliations:** 1 Neuroscience Institute, National Research Council (CNR), Padua, Italy; 2 Neuroscience Institute, National Research Council (CNR), Pisa, Italy; 3 European Laboratory for Non-Linear Spectroscopy (LENS), Sesto Fiorentino, Italy; 4 The Biorobotics Institute, Scuola Superiore Sant’Anna, Pisa, Italy; 5 Interdisciplinary Health Science Center, Scuola Superiore Sant’Anna, Pisa, Italy; 6 Bio@SNS Laboratory of Biology, Scuola Normale Superiore, Pisa, Italy; 7 Bertarelli Foundation Chair in Translational Neuroengineering, Center for Neuroprosthetics and Institute of Bioengineering, École Polytechnique Fédérale de Lausanne (EPFL), Lausanne, Switzerland; UCSD, UNITED STATES OF AMERICA

## Abstract

Stroke is a leading cause of long-term disability, frequently associated with persistent motor deficits. Gamma band oscillations, generated by synchronous discharge of parvalbumin-positive interneurons (PV-INs), are critically affected after stroke in humans and animals. Both gamma band and PV-INs play a key role in motor function, thus representing a promising target for poststroke neurorehabilitation. Noninvasive neuromodulatory approaches are considered a safe intervention and can be used for this purpose. Here, we present a novel, clinically relevant, noninvasive, and well-tolerated sub-acute treatment combining robotic rehabilitation with advanced neuromodulation techniques, validated in a mouse model of ischemic injury. During the sub-acute poststroke phase, we scored profound deficits in motor-related gamma band activity in the perilesional cortex. These deficits were accompanied by reduced PV-IN firing rates and increased functional connectivity, both at the perilesional and at the whole-cortex levels. Therefore, we tested the therapeutic potential of coupling robotic rehabilitation with optogenetic PV-IN-driven gamma band stimulation in a subacute poststroke phase during motor training to reinforce the efficacy of the treatment. Frequency-specific movement-related gamma band stimulation, when combined with physical training, significantly improved forelimb motor function. More importantly, by pairing robotic rehabilitation with a clinical-like noninvasive 40 Hz transcranial Alternating Current Stimulation, we achieved similar motor improvements mediated by the effective restoring of movement-related gamma band power, improvement of PV-IN maladaptive network dynamics, and increased PV-IN connections in premotor cortex. Our research introduces a new understanding of the role of parvalbumin-interneurons in poststroke impairment and recovery. These results highlight the synergistic potential of combining perilesional gamma band stimulation with robotic rehabilitation as a promising and realistic therapeutic approach for stroke patients.

## Author summary

Stroke-induced motor deficits are accompanied by alteration of gamma modulation and PV-IN network; all of these parameters are restored by noninvasive gamma stimulation and robotic therapy.

## Abstract 3

How can motor function be restored after stroke? This study shows in mice that combining robotic rehabilitation with non-invasive gamma band neuromodulation improves motor recovery by restoring movement-related oscillations and parvalbumin interneuron dynamics.

## Introduction

Stroke-induced functional deficits in the motor cortex represent one of the main causes of disability worldwide. Recently, poststroke physical therapy has been enriched by novel rehabilitative strategies, such as the combination of robotic tools with neuromodulatory techniques [1–3]. Robotic therapy is a promising tool for collecting highly precise kinetic and kinematic data about motor performance and providing customizable exercises. In addition, it is widely accepted that physical therapy is crucial for stroke rehabilitation but should be coupled with neuromodulatory strategies to maximize its effectiveness. These strategies aim to enhance plasticity and “prime” spared perilesional tissue, enhancing its susceptibility to “activity-dependent plasticity”. Such strategies can involve plasticizing drugs (i.e., Fluoxetine) or noninvasive brain stimulation (NIBS) techniques that enable precise and controlled modulation of cortical activity [4,5]. The optimal interplay between a plastic environment and proper physical exercises is essential for motor recovery [6].

Over the past decades, there has been a growing understanding of the importance of oscillatory neural activity in motor function in both healthy and injured subjects. Specifically, synchronized oscillations in the higher-gamma frequency range (60–90 Hz), leading to an increase in gamma power, coincide with movement initiation and execution (referred to as movement-related gamma synchronization) and reflect the initial activation of primary motor neurons involved in movement [7,8]. Studies in humans have demonstrated that driving higher-gamma oscillations enhances motor performance [9], demonstrating a causal role of these neural rhythms in plasticity and motor control [10,11]. Conversely, lower gamma band oscillations (30–60 Hz) are implicated in strategies for controlling stronger muscle force production [12].

In humans, an ischemic attack in the middle cerebral artery territory induces acute alterations of the neural rhythmic activity at rest in both affected and unaffected hemispheres [13]. Notably, an asymmetric enlargement of delta (2–3.5 Hz) and theta (4–7.5 Hz) band powers in the affected hemisphere accompanies ischemic events, while reduced gamma activity correlates with compromised hand functionality. Consistently, stroke survivors with incomplete upper-limb motor recovery and persistent deficits showed significantly lower gamma-band cortico-muscular coherence when performing a reach task using shoulder/elbow muscles [14]. This decreased coherence could reflect poor brain-muscle communication or poor integration of the signals from the two sources during motor actions. Such poor electroencephalography–electromyography coherence could reflect an underlying mechanism contributing to impaired reaching performance in stroke patients [14]. Conversely, increased gamma power in the affected hemisphere was associated with better recovery prospects [13]. Recent findings in animal models and human studies suggest a direct link between oscillatory activity in the gamma band and the excitatory/inhibitory balance within reciprocally connected networks of GABAergic interneurons (IN) and pyramidal cells within the primary motor cortex [15–17]. Indeed, Nowak and colleagues [18] demonstrated that driving gamma frequency oscillation via NIBS techniques is feasible and produces significant effects on motor function and GABA-mediated inhibition in healthy subjects.

In this context, the specific firing characteristics of a well-defined subgroup of IN, the fast-spiking parvalbumin-positive (PV) GABAergic IN, have demonstrated a causal role in generating and modulating gamma rhythms [16,19,20]. Recently, in rodent models for stroke, PV-IN activity has been linked to poststroke dysfunctions as well as motor recovery [21]. Accordingly, fast spiking IN optogenetic modulation has been used to improve motor recovery in animal models [22,23]. Importantly, Wang and colleagues demonstrated that perilesional optogenetic 40 Hz stimulation on inhibitory IN in acute phase after stroke increases neuronal survival and plasticity [22,24]. Rehabilitation appears to directly engage PV-INs, strengthening their synaptic connections and facilitating the reorganization of neuronal networks, possibly via increased gamma activity. These findings underscore the therapeutic potential of targeting PV-INs to enhance functional recovery after stroke [25].

This body of evidence may provide a good foundation for the development of new therapeutic strategies involving the modulation of brain rhythms in association with physical therapy. Despite these advancements, a coherent framework integrating and adapting these novel paradigms into human postrehabilitation strategies remains elusive. Optogenetic approaches, with their very restricted timing, must be properly translated to clinical realities for successful human application. This gap is also increased by the high variability among lesion volumes, infarct location, and clinical approaches [26]. Moreover, the precise neurophysiological mechanisms underlying the effects of functional damage and poststroke therapies are still poorly defined. As a result, despite rehabilitation, a significant percentage of stroke patients display persistent impairments in activities of daily living.

This study explores the longitudinal effects of the ischemic lesion on PV-INs and movement-related gamma band activity in a mouse model of ischemic stroke. Moreover, we used this data to validate the translational therapeutic potential of inducing gamma frequency oscillations in the spared forelimb premotor cortex combined with robotic-guided motor rehabilitation. We employed optogenetics to induce PV-IN-guided gamma rhythm in combination with daily exercise on a custom-made device designed for mouse forelimb training [27]. We then confirmed the efficacy of the treatment using a clinically relevant NIBS approach combined with robotic rehabilitation. Our data provide novel crucial insights into the neural mechanisms driving recovery and offer a reliable support for the development of more effective poststroke therapies.

## Results

### The ischemic lesion induces motor-related dysfunction in gamma band synchronization

**Fig 1:**
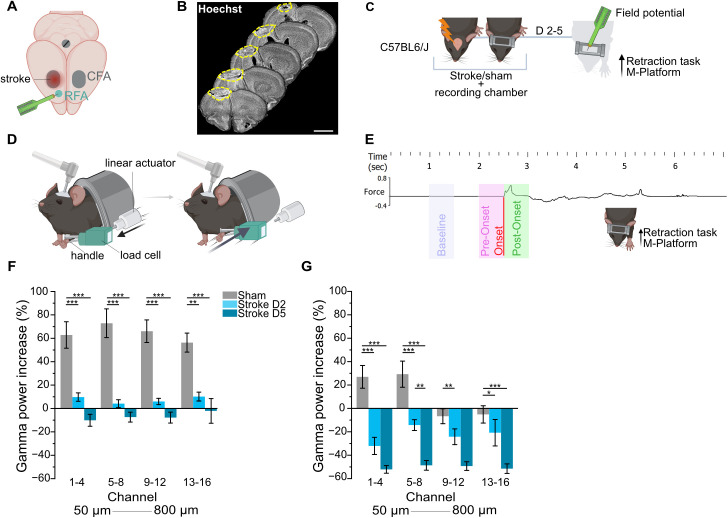
Impaired motor-related gamma band regulation after stroke. Electrophysiological recordings during voluntary movement in the perilesional premotor cortex revealed a significant deficit in gamma band modulation following ischemic stroke in the motor cortex. **A**, Schematic representation of the stroke lesion in the right CFA and the perilesional region (RFA) where the electrophysiological recordings were performed. **B**, Representative coronal brain slices of a stroke animal labeled with Hoechst nuclear staining showing the lesion (outlined by the dashed yellow line). Scale bar = 2 mm. **C**, Schematic representation of the experimental protocol. **D**, Schematic representation of the two different phases of the retraction task on the M-platform. Mice are head restrained with their wrist closed in a handle connected to the load cell for force detection. In the passive phase (left), the linear actuator pushes forward the handle and mouse forelimb. In the active phase (right), the mouse is trained to return the handle to the home position by overcoming a defined friction in order to receive a reward. **E**, Windows of analysis during the task in relation to movement onset. The black line represents an example of force trace throughout the task; the onset of the movement is highlighted in red; the purple window identifies the baseline period when no movement is detected; the pink window identifies the preonset phase (i.e., movement preparation) and the green window the postonset phase (i.e., movement). **F** and **G**, Quantification of gamma band power across all the 16 channels spanning all the cortical layers (channel 1 ≃ 50 μm, channel 16 ≃ 800 μm) in pre- and postonset windows, respectively. The graph presents data averaged over groups of four channels; for single-channel details, refer to S1C and S1D Fig. Healthy mice (Sham, gray, *n *=* *9), animals recorded 2 days after stroke (D2, light blue, *n *=* *6), mice recorded 5 days after stroke (D5, dark blue, *n *=* *8). The positive gamma band modulation in preonset and in the higher layers in postonset phase is abolished and even reversed in subacute stroke. Two-way ANOVA followed by Tukey test, * *P* < 0.05, *** *P* < 0.001. Data are shown as mean ± SEM. Created in BioRender. Vignozzi, L. (2025) https://BioRender.com/md8zh2c. The data underlying this figure can be found in https://data.mendeley.com/datasets/mw82tzp4rx/1.

We first assessed the impact of an ischemic lesion in Caudal Forelimb Area (CFA) on gamma band regulation in the spared perilesional premotor cortex (Rostral Forelimb Area, RFA) during voluntary movement. Mice had either undergone a photothrombotic lesion or sham surgery in the CFA (Fig 1A). A representative image of the stroke lesion is shown in Fig 1B (dashed yellow line). Motor function assessment was performed with the Gridwalk test before and after stroke, as shown in S1A Fig. Local field potential recordings from the RFA were taken while mice were engaged in a voluntary retraction task on the M-Platform, a custom-made apparatus for functional evaluation and neurorehabilitation of the forelimb in mice (Fig 1C and 1D). In order to fully sample the “early subacute phase,” preceding the glial scar formation [28], which is crucial for the beginning of rehabilitation protocols, recordings were performed at 2 and 5 days poststroke. We quantified gamma band power within specific time windows delineating a baseline (i.e., −1 to −1.5 s from movement onset), a preonset phase (PRE, from −500 ms to onset), and a postonset phase (POST, from onset to +500 ms) as illustrated in Fig 1E. In sham animals, we observed a significant increase in gamma band power in the premotor cortex in the preonset phase across all the cortical layers (Figs 1F and S1C, gray bar plot). Throughout movement execution (POST), gamma power remained elevated in the superficial layers (channel 1–8 of the linear multiprobe), while gamma modulation was minimally evident in deeper layers of the cortex (channel 9–16 of the linear multiprobe, Figs 1G and S1D, gray bar plots). In contrast, stroke animals assessed 2 (D2) and 5 (D5) days postlesion exhibited no gamma band upregulation before movement onset (Fig 1F, light blue and blue, respectively) and showed significantly lower gamma power during movement compared to controls. Consistently, during movement execution, gamma band power in stroke animals decreased across all cortical layers and resulted significantly different from controls in upper layers, where gamma band regulation was more pronounced (Figs 1G and S1D). Baseline gamma power did not differ between sham and stroke mice (S1B Fig).

### Ischemic stroke profoundly changes the resting state functional connectivity of PV-INs up to 1 month after injury

**Fig 2:**
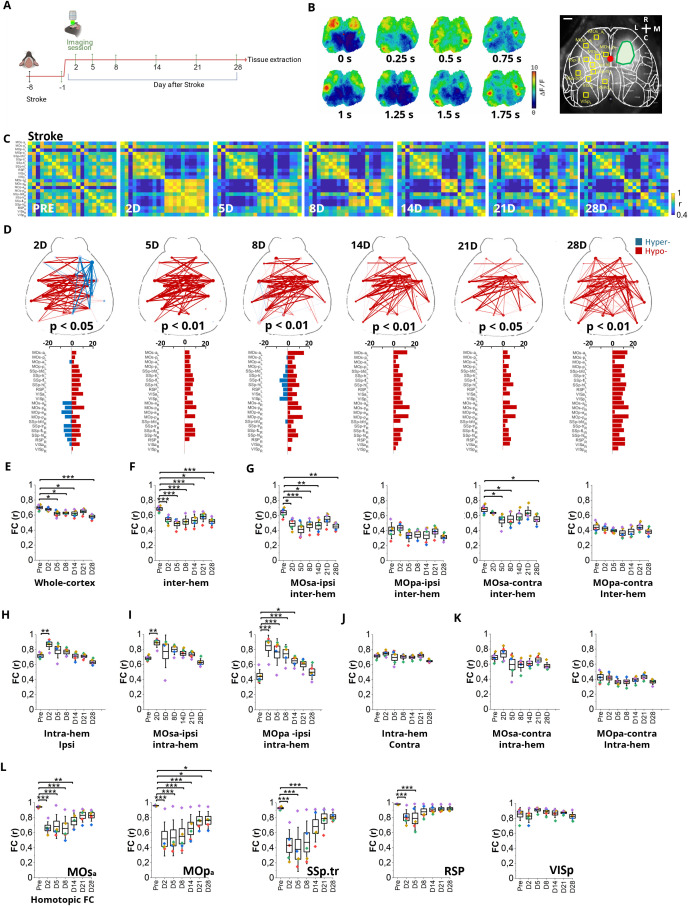
Enduring reduction of resting state functional connectivity (FC) of parvalbumin-positive interneurons (PV-INs) after stroke. **A,** Experimental timeline, with the cranial optical window implanted one week before the first imaging session. Imaging time point at −1 (PRE), 2, 5, 8, 14, 21, and 28 days after stroke. **B,** Left, representative image sequence of cortical PV-IN activity before stroke. The black dot indicates bregma. L: lateral; M: medial; R: rostral; C: caudal (Scale bar, 1 mm). Right, wide-field calcium imaging field-of-view aligned with the surface of the Allen Institute Mouse Brain atlas. The green area on the left hemisphere locates the damaged region. Yellow squares represent cortical areas defined in both left (L, contralesional) and right (R, ipsilesional) hemispheres. Red dot indicates bregma (Scale bar, 1 mm). **C**, Pairwise Pearson’s correlation coefficients of cortical activity were visualized as averaged correlation matrices for each imaging time point after hemodynamic correction. **D**, Network diagrams of statistically significant FC alterations after 2, 5, 8, 14, 21, or 28 days from injury. Blue and red lines denote significant hyper-correlation and hypo-correlation compared to prestroke values, respectively. The bar plots (bottom) indicate the number of significant FC alterations for each cortical area. **E**, Box chart illustrating FC averaged over the whole cortex. **F**, Box chart illustrating the averaged inter-hemispheric FC. **G**, Box charts showing inter-hemispheric FC of ipsilesional and contralesional secondary and primary motor cortices in the anterior regions (MOsa and MOpa, respectively). **H**, Intra-hemispheric FC of ipsi-lesional areas. **I**, Box charts displaying intra-hemispheric FC of the secondary (left) and primary (right) ipsilesional motor cortices in the anterior region. **J**, Intra-hemispheric FC of contralesional areas. **K**, the box charts displaying intra-hemispheric FC of the secondary (left) and primary (right) contralesional motor cortices in the anterior region. **L**, Homotopic FC changes from prestroke to 28 days after injury (MOsa, anterior secondary motor cortex; MOsp, anterior primary motor cortex; SSp.tr, primary somatosensory cortex-trunk; RSP, dorsal part of the retrosplenial cortex; VISp, primary visual cortex). One-way ANOVA followed by Tukey test, * *P* < 0.05, ** *P* < 0.01, *** *P* < 0.001. Data are shown as mean ± SEM. Each color indicates a single subject, *n* = 5. Created in BioRender. Vignozzi, L. (2025) https://BioRender.com/md8zh2c. The data underlying this figure can be found in https://data.mendeley.com/datasets/mw82tzp4rx/1.

We reasoned that the impairment of gamma band regulation could be associated with local and widespread changes in the functionality of PV-INs. To test this hypothesis, we performed longitudinal wide-field calcium imaging in awake head-fixed mice expressing GCaMP7f in PV-INs (Figs 2A, S2G, and S2H). We recorded spontaneous PV-IN activity one day before (PRE-STROKE) and at 2, 5, 8, 14, 21, and 28 days after stroke in the same mice, monitoring the evolution of the lesion from the acute to the chronic phase (i.e., 30 days in mice). In line with electrophysiological experiments, the “early subacute phase” after stroke was sampled with particular emphasis considering the importance as a sensitive time window for the beginning of the rehabilitation protocol. Spontaneous PV-IN activity underwent a drastic reduction in the peri-infarct cortex, involving a large fraction of both hemispheres (Fig 2C). We therefore hypothesized major alterations in PV-IN functional connectivity (FC) after the lesion. Thus, FC of the entire dorsal cortex was evaluated by computing Pearson’s correlation (Fisher’s z-transformed) between all paired cortical regions after hemodynamic correction (Fig 2B). In healthy mice, resting state FC was homogeneous and symmetrical across hemispheres. Notably, the anterior primary motor cortices displayed low correlation values, with the sole exception being the homotopic connectivity. In the sub-acute phase after stroke, marked alterations were evident, with opposite consequences on intra- and inter-hemispheric FC (Fig 2C). Although far from prestroke condition, the average FC became more homogeneous during the following weeks. This qualitative assessment was confirmed by computing differences between pre- and poststroke FC across timepoints (S2A Fig) and testing for significant changes in correlations using the network-based statistic [29,30] (Fig 2D). Interestingly, a hypo-connected interhemispheric network emerged early and persisted up to 28 days after stroke. In contrast, the intra-hemispheric FC of the lesioned side showed a transient increase in the peri-infarct regions during the sub-acute phase followed by a gradual reduction in the chronic phase. One month after stroke, the hypo-correlated network expanded over the entire dorsal cortex, involving also distant regions like visual areas (Fig 2D). By quantifying the whole-cortex FC across time, we confirmed that the global reduction was not recovered 28 days after stroke (Fig 2E). Then, the contribution of inter- and intra-hemispheric FC to the global decrease was evaluated separately. Interestingly, inter-hemispheric connectivity was dominated by a decrease in correlation strength (Fig 2F). Notably, the anterior portion of the ipsilesional secondary motor cortex (MOsa) is the only motor area to display a significant FC decrease following stroke (Fig 2G). Instead, intra-hemispheric FC shows a large but transient increase ipsilateral to the injury site (Fig 2H), with a strong contribution from both the anterior portion of the peri-infarct primary and secondary motor cortices (Fig 2I). Instead, there were no significant changes in the contralateral intra-hemispheric FC (Fig 2J and 2K). Overall, these results imply that the decrease in global strength is mostly brought on by inter-hemispheric alterations. Therefore, we specifically dissected the contribution of homotopic desynchronization: substantial decrease of homotopic connectivity was primarily affecting the peri-infarct and associative regions (M2, M1, SSp.tr, RSP) in the sub-acute phase, followed by a partial recovery starting a week from the damage (Fig 2L). The quantification of the ischemic lesion volume and of motor performance in Gridwalk is shown in S2F and S2G Fig. Our results suggest profound alterations in PV-IN connectivity, which are not restricted to the injured site but extend throughout the entire dorsal cerebral cortex, primarily affecting inter-hemispheric strength.

To assess the potential influence of global activity patterns on FC, we performed the same analysis after applying global signal regression (GSR, S4 Fig). As expected by removing widespread global components, GSR shifted the overall FC values toward lower correlations across the cortex. However, global network alterations after stroke were consistent with those observed without GSR (S4C Fig). We observed a persistent reduction of inter-hemispheric FC (S4D and S4E Fig) and a transient increase of intra-hemispheric FC ipsilateral to the lesion (S4F Fig). Similarly, homotopic connectivity across peri-infarct and associative regions showed an early decrease with partial recovery at later stages (S4J Fig). Notably, GSR revealed a persistent impairment of intra-hemispheric connectivity between peri-infarct regions (S4G Fig), which remained significantly impaired across all poststroke time points.

Our findings highlight that while FC measured without GSR may suggest widespread compensatory processes, the recovery of local functional networks remains severely limited in the peri-infarct cortex, even 1 month after injury by indicating a failure of spontaneous recovery in the chronic phase.

Furthermore, to explore the relationship between PV-based FC and hemodynamic signaling, we performed a parallel FC analysis using the 530 nm reflectance signal, which primarily reflects changes in hemoglobin concentration. Interestingly, we observed a strong correspondence between calcium-based PV FC maps (Fig 2C) and those derived from hemodynamic signals (S7D Fig). This similarity is consistent across both healthy and poststroke conditions. These results are in line with previous findings showing that GCaMP signals from excitatory neurons can recapitulate hemodynamic-based FC maps [31,32] and extend this concept to PV+ inhibitory neurons. Importantly, this observation suggests that PV-based calcium imaging may capture network-level dynamics that are ultimately reflected in the slower hemodynamic fluctuations measured by techniques like fMRI and Intrinsic Optical Signal Imaging.

### PV-INs in perilesional premotor cortex involved in motor execution can be engaged by optogenetic stimulation

**Fig 3:**
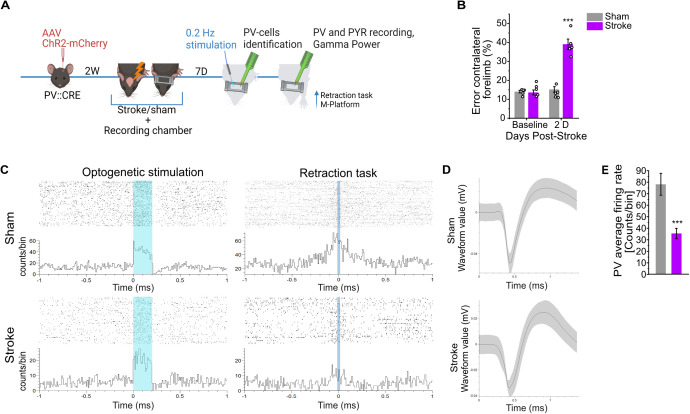
Parvalbumin-positive interneurons (PV-INs) in perilesional tissue respond to optogenetic stimulation and are involved in voluntary movement, but show a decreased firing rate. Optogenetic stimulation and single-unit recordings were applied in order to identify PV-INs in the premotor cortex of healthy and ischemic animals and evaluate their discharge properties and their involvement in a voluntary movement. **A**, Schematic representation of the experimental procedure is reported. **B**, Motor function assessment in the Gridwalk test before and 2 days after stroke induction in sham (*n *=* *5, gray bar plots) and stroke (*n *=* *5, purple bar plots) animals, circles represent single animals (two-way ANOVA followed by Tukey test *** *P* < 0.001). **C**, Representative raster plots of identified PV-INs for sham and stroke animals. Upper panels display the raster plot of a PV-IN in response to optogenetic stimulation (left) and temporally aligned with movement onset (right) in a sham animal. Lower panels present the same raster plots for a stroke animal. **D**, Average waveforms of recorded PV-INs for sham and stroke animals. **E**, Quantification of the average firing rate of the recorded PV-INs in response to optogenetic stimulation. A significant decrease in firing activity is evident in stroke animals (Two-tailed *T* Test *** *P* < 0.001). Data are shown as mean ± SEM. Created in BioRender. Vignozzi, L. (2025) https://BioRender.com/md8zh2c. The data underlying this figure can be found in https://data.mendeley.com/datasets/mw82tzp4rx/1.

Once the significant impact of ischemic injury in the CFA on PV-IN activity in perilesional tissue was established, we investigated whether the PV-IN circuitry involved in voluntary movement could still be engaged by external stimulation. We induced either an ischemic or a sham lesion in CFA of B6;129P2-Pvalb tm1(cre)Arbr/J (PV-CRE) mice previously injected with a dflox.hChR2 AAV in RFA. Motor function was evaluated with Gridwalk test before and D2 after lesion in order to assess motor impairment in stroke mice (Fig 3B). Subsequently, recordings from the spared perilesional premotor cortex were conducted 5–7 days after ischemic or sham lesion in awake, head-restrained mice. The time window was chosen considering the beginning of a neuromodulation phase starting from day 5. First, we used 200 ms blue-light pulses to identify putative PV-IN that exhibited increased firing rate lasting for the entire duration of the stimulation and putative neurons connected to the stimulated PV-IN, whose spontaneous discharge was inhibited during the stimulation. Next, we monitored the identified neurons during the execution of the forelimb retraction task on the M-Platform (Fig 3A). Out of 35 PV-INs identified in 6 healthy animals, 19 correlated positively or negatively with movement onset. In 4 stroke animals, 17 out of 34 putative PV-INs were movement-related (Fig 3C and 3D). PV-INs in stroke animals exhibited a significantly reduced firing rate during stimulation (Fig 3E). These findings demonstrated that, despite the profound impairment of PV-IN network and cell responsiveness resulting from the ischemic lesion, individual cells retain their reactivity, their involvement in forelimb movement execution and could be recruited by external stimulation.

### Selective 40 Hz optogenetic PV-IN stimulation combined with robotic rehabilitation restores forelimb function

**Fig 4:**
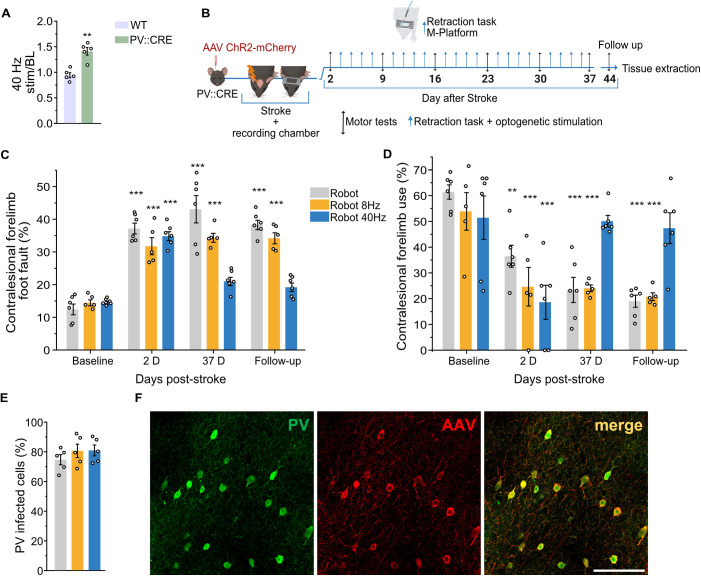
Robotic rehabilitation combined with optogenetic parvalbumin-positive interneuron (PV-IN) stimulation at 40 but not 8 Hz improves motor function after stroke. **A**, Average Gamma power response across all the cortical layers (i.e., the average of the values detected by the 16 channels of the linear electrode) to a 40 Hz optogenetic stimulation in PV-CRE mice (green, *n *=* *5) but not in WT mice (pink, **n *= 5*) infected with dflox.hChR2 AAV (Student *t* test, ** *P < 0.01*). **B**, Schematic of the experimental protocol. **C** and **D**, Forelimb motor function assessment in Gridwalk and Schallert cylinder test respectively. Robotic rehabilitation alone (gray bar plots, *n* =* *6) is not able to restore forelimb motor function as expected. Similarly, 8 Hz stimulation (yellow bar plot, *n *=* *5) fails to provide any improvement to motor rehabilitation. On the contrary, 40 Hz stimulation (blue bar plots, *n *=* *6) significantly improved motor function. Two-way RM ANOVA followed by Tukey test, * *P* < 0.05 ** *P* < 0.01, *** *P* < 0.001. **E**, Quantification of PV-infected cells in the perilesional premotor cortex in the three experimental groups, revealing no significant differences among them (Robotic rehabilitation alone, *n *=* *5; 8 Hz stimulation, *n *= 5; 40 Hz stimulation, *n *= 5). One-way ANOVA followed by Dunnett’s test, *P* > 0.05. **F**, Representative image of PV-INs (in green), AAV-infected cells (in red). Scale bar: 100 µm. Data are shown as mean ± SEM, circles represent single animals. Created in BioRender. Vignozzi, L. (2025) https://BioRender.com/md8zh2c. The data underlying this figure can be found in https://data.mendeley.com/datasets/mw82tzp4rx/1.

In order to determine the therapeutic potential of PV-IN-driven gamma band stimulation, we used optogenetic stimulation in PV-CRE mice previously injected with a dflox.hChR2 AAV in RFA. We first demonstrated that 40 Hz optogenetic stimulation (3 s pulses) increases gamma band power in PV-CRE mice, but not in WT mice injected with dflox.hChR2 AAV, thus confirming the direct involvement of PV-IN activation in gamma band generation (Fig 4A). Subsequently, we investigated a combined neurorehabilitative approach where PV-CRE mice injected with dflox.hChR2 AAV were subjected to photothrombotic stroke in CFA. Mice were divided into three experimental groups: the Robot group received sham stimulation, the Robot 8 Hz group was treated with 8 Hz optogenetic stimulation in RFA during retraction task execution, and the Robot 40 Hz group received 40 Hz stimulation. All groups received daily robotic rehabilitation on the M-Platform. All the treatments were applied starting from day 5 poststroke and were applied until 37 days after stroke (chronic phase). Treatment efficacy was evaluated using the Gridwalk and Schallert Cylinder test, performed at baseline, 2 days after stroke (pretreatment), once a week until 37 days poststroke and after 1 week of follow-up without treatment. The experimental protocol schematic is shown in Fig 4B. Initial deficits detected with both behavioral motor tests were consistent across groups (Fig 4C and 4D). Robotic rehabilitation alone (gray bar plots) did not yield therapeutic benefits even after 5 weeks of treatment. Of note, combining 8 Hz stimulation with Robotic rehabilitation (yellow bar plots) did not lead to additional motor improvement, indicating that PV-IN activation at a different oscillatory pattern fails to restore motor function. Conversely, 40 Hz stimulation combined with robotic rehabilitation (blue bar plots) resulted in a highly therapeutic effect, evident both in the number of contralesional forelimb foot faults of the Gridwalk test and in the percentage of contralesional forelimb use of the Schallert Cylinder test. In both functional motor tests, the parameters reverted to baseline levels 37 days after injury. Importantly, the improvement remained stable during the follow-up assessment conducted one week after treatment conclusion. Postmortem analyses showed no significant differences in PV-infected cells percentages among groups (Fig 4E). In Fig 4F, a magnification of the perilesional RFA of the Robot 40 Hz group is reported as an example of double labeling of AAV-infected cells (in red) and immunohistochemically labeled PV-INs (in green).

### Noninvasive gamma band stimulation in perilesional premotor cortex coupled with robotic rehabilitation improves forelimb motor function after stroke

**Fig 5:**
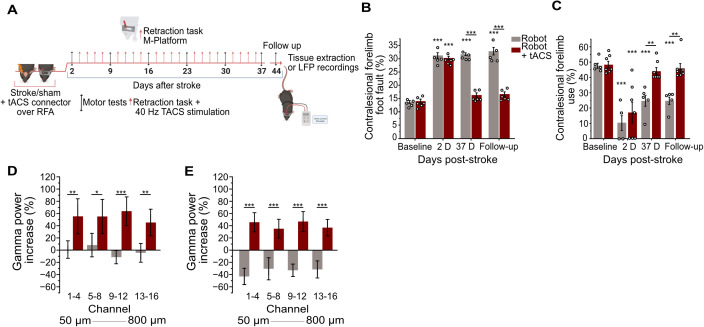
Forelimb motor improvements can be achieved by coupling robotic rehabilitation with noninvasive 40 Hz transcranial Alternating Current Stimulation (tACS). **A,** Schematic of the experimental protocol. **B** and **C**, Motor function assessment in mice treated with robotic rehabilitation alone (gray bar plots, *n *=* *5) and coupled with noninvasive 40 Hz tACS (red bar plots, *n *=* *6) on the perilesional premotor cortex evaluated with Gridwalk test and Schallert Cylinder test, respectively. The use of 40 Hz stimulation succeeded in restoring forelimb motor function and maintained this improvement in a follow-up measure. **D** and **E,** Quantification of gamma band power in pre- and postonset windows, respectively, across all 16 channels spanning the entire cortical layers at the follow-up time point (channel 1 ≃ 50 μm, channel 16 ≃ 800 μm). The graph presents data averaged over groups of four channels; for single-channel details, refer to S3C and S3D Fig. Gray bar plots *n *=* *7, red bar plots *n *=* *6. Two-way RM ANOVA followed by Tukey test, * *P* < 0.05 ** *P* < 0.01, *** *P* < 0.001. Data are shown as mean ± SEM, circles represent single animals. Created in BioRender. Vignozzi, L. (2025) https://BioRender.com/md8zh2c. The data underlying this figure can be found in https://data.mendeley.com/datasets/mw82tzp4rx/1.

We then evaluated whether the therapeutic effect obtained with specific optogenetic activation of PV-INs could be replicated using a more translational approach, the transcranial Alternating Current Stimulation (tACS). To this end, a photothrombotic stroke was induced in the CFA of a total number of 12 C57Bl6/J mice, which were subsequently divided into two experimental groups. All animals underwent 5 weeks of daily robotic rehabilitation, in line with the previous experiment. The Robot tACS group received 40 Hz tACS applied over the perilesional RFA, while the Robot group underwent sham stimulation, entailing electrode insertion and connection without actual current transmission (Fig 5A). Motor function, assessed using Gridwalk and Schallert Cylinder tests, revealed significant deficits 2 days after the ischemic lesion induction, while no differences were observed between Robot tACS group and Robot group before lesion and D2 postlesion. Robotic rehabilitation alone did not lead to recovery. However, the Robot tACS group displayed significant improvements, returning to baseline in both tests and maintaining these gains during the follow-up period (Fig 5B and 5C).

Next, we evaluated whether tACS treatment could induce a recovery in gamma band oscillations in a new cohort of C57Bl6/J mice subjected to photothrombotic stroke in the CFA. Mice were divided into two experimental groups: the Robot-tACS group underwent 40 Hz tACS combined with robotic rehabilitation, while the Robot group received robotic rehabilitation alone. At the follow-up time point, the Robot-tACS group exhibited a significant increase in gamma band power in both the pre- and postonset windows (Figs 5D, 5E, S3C, and S3D), suggesting that the combined treatment approach not only improves motor function but also has a substantial impact on power within the gamma frequency range. Quantification of lesion volume showed no differences among animals untreated and treated with robot or robot + tACS (S3A Fig). Motor performance assessed with Gridwalk test confirmed motor recovery for this robot + tACS group with respect to both untreated and robot-only groups (S3B Fig). These findings underline the potential benefits of integrating robotic therapy with tACS in order to promote neural recovery and rehabilitation.

### Adaptive contralesional reorganization of PV-INs FC drives motor recovery after stroke in rehabilitated mice

**Fig 6:**
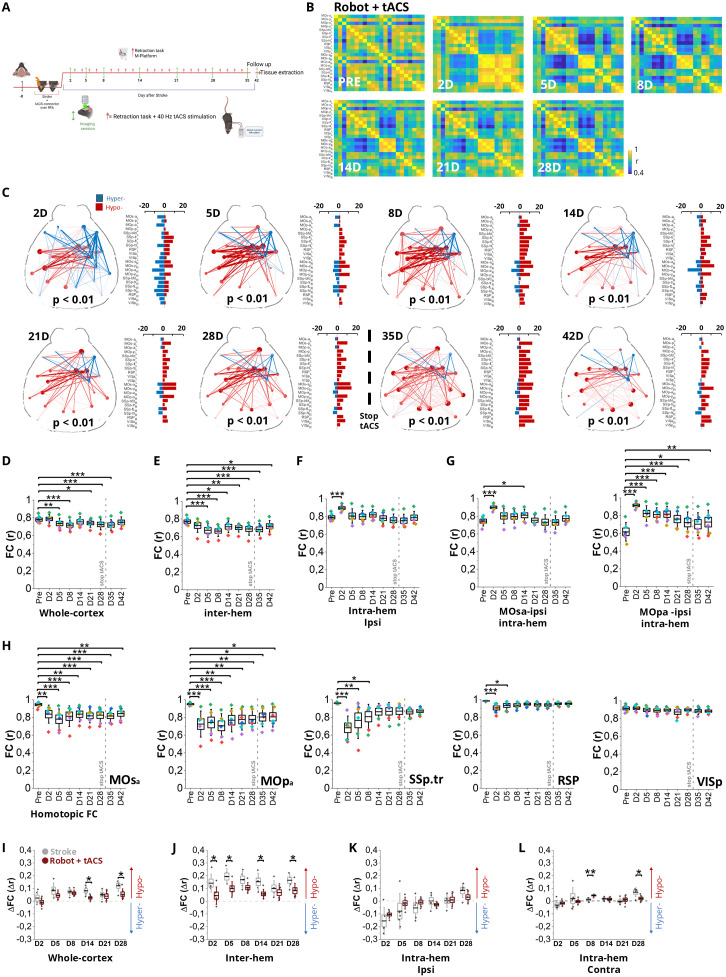
Combined rehabilitation improves maladaptive network dynamics. **A,** Experimental timeline, with the cranial optical window implanted one week before the first imaging session. Imaging time point at −1 (PRE), 2, 5, 8, 14, 21, and 28 days after stroke. tACS was performed from day 2 to day 28 followed by two time points of follow-up (day 35 and day 42). **B,** Pairwise Pearson’s correlation coefficients of cortical activity were visualized as averaged correlation matrices for each imaging time point prestroke and 2, 5, 8, 14, 21, or 28 days after injury. **C**, Network diagrams of statistically significant FC alterations after 2, 5, 8, 14, 21, 28, 35, and 42 days from injury. Blue and red lines denote significant hyper-correlation and hypo-correlation compared to prestroke values, respectively. The bar plots (on the right) indicate the number of significant FC alterations for each cortical area. **D**, Box chart illustrating FC averaged over the whole cortex. **E**, Box chart illustrating the averaged inter-hemispheric FC. **F**, Intra-hemispheric FC of ipsi-lesional areas. **G**, Box charts displaying intra-hemispheric FC of the secondary (left) and primary (right) ipsilesional motor cortices in the anterior region. **H**, Homotopic FC changes from prestroke to 28 days after injury (MOsa, anterior secondary motor cortex; MOsp, anterior primary motor cortex; SSp.tr, primary somatosensory cortex-trunk; RSP, dorsal part of the retrosplenial cortex; VISp, primary visual cortex). One-way ANOVA followed by Tukey test, * *P* < 0.05, ** *P* < 0.01, *** *P* < 0.001. Data are shown as mean ± SEM. The gray dashed line indicates the beginning of the follow-up period after the end of tACS treatment. Each color indicates a single subject, *n* = 5. **I-L** Comparison of changes in functional connectivity (ΔFC) relative to baseline (prestroke) between Stroke (gray) and Robot + tACS (dark red) groups across different time points after stroke (D2–D28) in terms of whole-cortex **(I)**, inter-hemispheric connectivity **(J)**, intra-hemispheric connectivity of ipsilesional hemisphere (**K**), and intra-hemispheric connectivity of the contralesional hemisphere **(L)**. Data are shown as box plots (mean ± SE). *p < 0.05, main effect of treatment (two-sample *t* test). Blue and red arrows indicate the directions of hyper- and hypo-connectivity compared to the prestroke FC, respectively. Created in BioRender. Vignozzi, L. (2025) https://BioRender.com/md8zh2c. The data underlying this figure can be found in https://data.mendeley.com/datasets/mw82tzp4rx/1.

To further investigate the mesoscale mechanisms underlying motor recovery, we next analyzed large-scale cortical FC following combined rehabilitation. One week before stroke induction, mice were implanted with an optical window. A small opening was left in the cranial implant over the RFA to allow daily transcranial stimulation. Baseline wide-field calcium imaging was performed one day before stroke induction. Starting from day 2 after stroke, animals underwent daily sessions of robotic rehabilitation on the M-Platform, coupled with 40 Hz tACS. After 1 month of rehabilitation, 2 weeks of stimulation-free follow-up were executed (Figs 6A and S8).

We evaluated alteration in FC over weeks (Figs 6B and S2B) by computing differences between pre- and poststroke FC across timepoints after hemodynamic correction (S2C Fig). Interestingly, we observed a different network reorganization compared to spontaneous recovery in the late phase after combined rehabilitation. Initially (at 2 and 5 days after stroke), a hypo-connected network was still evident, similar to the untreated condition (S2C Fig). From day 8, the extent and strength of hypo-connectivity progressively diminished (S2C Fig). By day 28, before the end of the rehabilitation protocol, the hypo-connected network was largely attenuated, and after cessation of tACS (day 35 and 42), network alterations were further normalized (Fig 2C), with values continuing to rise during the follow-up period. Notably, whole-cortex FC was no longer significantly different from prestroke levels at Day 42, suggesting a sustained and consolidating effect of the combined intervention (Fig 6D). However, inter-hemispheric FC remains strongly altered (Figs 6E and S2D). Intra-hemispheric connectivity within the ipsilesional and contralesional hemisphere instead did not show significant alterations over time except for poststroke day 2 and poststroke day 5, respectively (Figs 6F, S2E, and S2F). Nevertheless, FC within the ipsilesional primary motor area (MOpa-ipsi, Fig 6G) remained persistently impaired up to 42 days poststroke, with no significant recovery even after the cessation of tACS stimulation, in contrast to the partial restoration observed in untreated animals.

Despite the overall improvement in global FC, homotopic FC did not exhibit a comparable recovery after combined robotic rehabilitation and tACS (Fig 6H). In particular, connectivity between MOsa remained significantly impaired throughout the observation period (Fig 6H). This result is in contrast with spontaneous stroke recovery, where a partial normalization of homotopic FC was observed over time.

To further explore the impact of rehabilitation, we performed a direct comparison between animals undergoing combined rehabilitation and stroke controls. Animals receiving robot rehabilitation combined with tACS showed a significant reduction in abnormal FC across the whole cortex (Fig 6I), in inter-hemispheric FC (Fig 6J), and in the intra-hemispheric contralesional FC (Fig 6L) 28 days after the stroke. In contrast, no major differences were observed between groups in the intra-hemispheric ipsilesional FC (Fig 6K), which was the direct target of the stimulation. This lack of modulation might reflect the severe disruption of ipsilesional circuits after stroke, which limits their capacity for functional recovery.

Previous studies have shown that removing the global component of functional signals can help uncover network alterations that might otherwise be obscured by widespread activity patterns. Interestingly, after removing the global component, FC alterations remained largely consistent with those shown without GSR (S5 Fig), with one notable exception. Intra-hemispheric FC within the contralesional hemisphere remained persistently elevated and failed to normalize over time (S5K, S6D, and S6E Figs). These results indicate that the functional motor recovery promoted by robotic rehabilitation and tACS (S2J Fig) does not correspond to a restoration of the prestroke network organization but instead relies on a persistent reorganization within the contralesional hemisphere to compensate for deficits caused by the infarct (S2I Fig).

### Combined neurorehabilitation increases PV-INs connections and modulates GABAergic system

**Fig 7:**
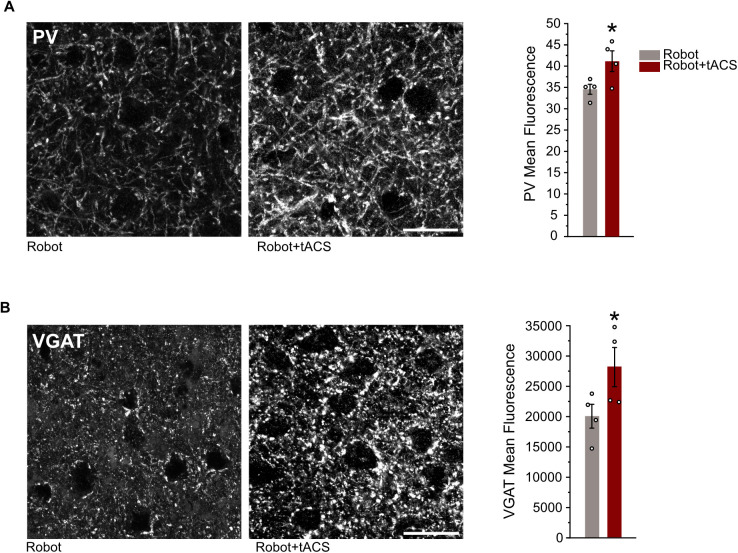
Increased parvalbumin-positive interneuron (PV-IN) connections and modulation of GABAergic transporters after combined neurorehabilitation. Immunohistochemical characterization of the consequences of combined neurorehabilitation on the density of PV-IN connections and the expression of GABAergic transporters. **A**, Magnification of the perilesional premotor cortex immunostained with anti-PV for robot-only and robot + tACS groups together with the quantification of mean anti-PV fluorescence calculated in puncta-rings around cell bodies of nonPV positive neurons (unpaired Student *t* test, * **P* *< 0.05). Scale bar: 20 µm. **B**, Magnification of the perilesional premotor cortex immunostained with anti-VGAT antibody for robot-only and robot+tACS groups together with the quantification of mean anti-PV fluorescence calculated in puncta-rings around cell bodies of VGAT positive neurons (unpaired Student t *t*est, * *P* < 0.05). Scale bar: 20 µm. Data are shown as mean ± SEM. The data underlying this figure can be found in https://data.mendeley.com/datasets/mw82tzp4rx/1.

Following the assessment of motor function, brain tissues from both Robot and Robot-tACS not implanted groups were collected for immunohistochemical analysis in order to explore structural and biochemical impacts of the treatments. Focusing on the spared perilesional premotor cortex, we investigated changes in PV-IN synaptic connections by examining PV expression in “puncta rings” surrounding nonPV neuronal somas. The Robot-tACS group exhibited increased mean fluorescence compared to the Robot group, indicating enhanced PV-IN connectivity (Fig 7A). Furthermore, we assessed whether the 40 Hz stimulation and the consequent increase in PV connections induces homeostatic changes in the GABAergic transporters in the perilesional cortex. We quantified the mean fluorescence of inhibitory terminals impinging on the soma of identified neurons. The expression of Vesicular GABA Transporter (VGAT) in the Robot-tACS group was significantly increased compared to Robot mice (Fig 7B). These findings reveal that 40 Hz tACS stimulation significantly impacts the PV-IN network and the GABAergic inhibitory system posttherapy.

## Discussion

In humans and nonhuman primates, lesions to the primary motor cortex induce extensive reorganization of regions adjacent to the infarct, such as premotor areas [33,34] and are thought to be related to motor (positive or negative) compensation. Despite the subdivision of primary motor cortex and premotor areas in mice is more subtle than in primates [35,36], two motor areas specific for the forelimb have been identified: the CFA and the RFA [37,38]. The RFA is thus to be considered a promising target for poststroke recovery in mice as the premotor areas in humans, as demonstrated by previous studies [39,40].

In this work, we explored the role of PV-IN networks and movement-related gamma modulation in poststroke motor damage and rehabilitation in mice, offering novel insights that could significantly boost poststroke treatment.

Our findings indicate that, following a stroke in the CFA, gamma modulation in the spared premotor cortex (RFA) is significantly altered both before and during voluntary movement. Additionally, ischemic lesions lead to pronounced impairment in both intra- and inter-hemispheric PV-IN connectivity, which worsens over time.

First, we recorded cortical activity in the perilesional RFA during a forelimb retraction task and observed gamma power modulation consistent with human studies [41–43]. Oscillatory gamma activity effectively engages brain areas during cognitive task and has been recently associated with motor control and execution [41–46].

This supports the premotor cortex’s role in movement planning and execution. Our results show increased gamma power across all cortical layers during the premovement window, reflecting the recruitment of the premotor cortex in preparing and planning the movement. During movement execution, gamma activity remained elevated in the higher layers, while the lower layers exhibited no significant modulation compared to the nonmovement condition. This layer-specific pattern suggests that the supragranular layers, likely involved in computational processing, differ functionally from the deeper layers, which house larger pyramidal neurons responsible for conveying movement signals to subcortical areas [47,48]. A similar layer-specific pattern has been described in the visual system, where gamma band power is largely confined in layer 4, contributing to encode visual contrast [49,50].

In general, layer-specific electrophysiological recordings indicate that gamma synchronization underlies feed-forward communication between cortical areas—primarily through supragranular layers, which are the main source of feed-forward projections [51]. Importantly, gamma band synchronization seems far from being a purely sensory-driven phenomenon, but it reflects a general aspect of cortical function [52], involving also inter-regional processing in motor areas. After stroke, gamma band modulation in the motor cortex was significantly impaired, both before and during movement execution. Remarkably, no distinctions were observed between stroke and sham mice in the baseline window, underscoring that the deficits in frequency modulation only emerge during active movement.

To correlate these findings with PV-IN connectivity alterations, we employed longitudinal Wide-Field imaging in PV-CRE mice infected with a viral vector expressing a floxed calcium indicator. Our results indicated significant disruption in PV activity and connectivity during the acute phase, primarily in peri-infarct cortical regions, with global hypoconnectivity persisting into the chronic phase of spontaneous recovery. Previous works using wide-field calcium imaging have underscored the crucial role of PV-INs in motor learning [44,45]. Despite their sparsity, inhibitory cells play a predominant role in many key brain functions, including neural network coordination [53] and memory formation [54]. Recent studies demonstrated a loss of homotopic connectivity in stroke animals [55–57]. Here, we reveal the role of IN in intra- and inter-hemispheric diaschisis by showing that PV-IN FC over the entire cortex is strongly compromised by a focal lesion, primarily on peri-infarct and homotopic regions. Stroke-induced de-differentiation of cortical activation may explain the transient increase in ipsilesional connectivity in the sub-acute phase. The reduced specificity in functional activation [58] might be advantageous in stroke subjects to activate brain regions nearby the lesion when executing functions that would normally require the damaged area. Therefore, hyperconnectivity could result from synchronous activation of the entire lesioned cortex. Dedifferentiation and synchronous activation of the entire cortex have already been shown to occur in excitatory cortical neurons during both acute and chronic phases in spontaneously recovering stroke mice [1]. Collectively, these findings suggest that the excitatory/inhibitory balance is significantly disrupted at both local and distal levels, beginning in the acute phase and stabilizing in the chronic phase. Our group previously demonstrated that poststroke rehabilitation can alter the FC of excitatory neurons [1]. Furthermore, recent work has highlighted the cortex-wide properties of PV-IN networks in a rat model of epilepsy [59], and our current research contributes further to this knowledge by addressing large-scale activity of PV-INs poststroke. Given the crucial role of inhibitory neurons in other neurological conditions, including autism and Alzheimer’s disease [60,61], we foresee that the same approach could be applied to study large-scale alterations of inhibitory circuits in these and other neuropathologies.

We explored whether impairments in PV-IN connectivity could contribute to the observed deficits in gamma band modulation. It has been demonstrated that PV-INs play an active role in voluntary movement execution through inhibition of pyramidal neurons [62,63], and dysfunction in cortical PV-IN networks has been reported in motor pathologies [64–67]. Combined with our wide-field imaging results, these findings suggest an early and long-lasting PV-IN network dysfunction, potentially affecting their recruitment as targets for neurostimulation. Using optogenetic stimulation and single-unit recordings in PV-CRE head-fixed mice, we identified PV-IN activity during voluntary movement. Isomura and colleagues [62] identified representative fast-spiking IN as PV-INs correlated with lever pulling in mice. More recently, Giordano and colleagues [68] demonstrated that fast-spiking IN in the motor cortex and RFA display unique discharge properties, firing earlier and longer during movement compared to pyramidal neurons. Accordingly, we found many cells responding to optogenetic stimulation manifesting spiking activity aligned with movement onset in both control and stroke animals. This confirms active, movement-related PV-INs in the perilesional premotor cortex that can be recruited for movement-related gamma band stimulation shortly after stroke. However, the ischemic lesion led to a significant reduction in the physiological spiking activity of these cells, as evidenced by a decrease of firing rate identified in our recordings. This aligns with our previous findings of decreased numbers of PV-INs and their connections impinging on pyramidal neurons in the perilesional cortex of chronic stroke animals [2,3,69,70]. This underscores the importance of timing in interventions, confirming a critical window for cortical plasticity [71–73] that has to be exploited for rehabilitative purposes.

Notably, despite the reduced firing rate in perilesional tissue, the surviving PV-INs remain responsive to optogenetic stimulation, with their discharge reliably correlated with voluntary movement. These findings suggest that even compromised PV-IN networks can still be recruited to trigger gamma oscillatory activity tuned with movement control, if properly stimulated during physical rehabilitation. In line with this hypothesis, the therapeutic potential of targeting gamma oscillations is increasingly explored [23,24,74].

Building on these observations, we first explored a novel neurorehabilitative approach, combining physical robotic therapy with 40 Hz optogenetic stimulation of PV-INs in spared perilesional tissue. Our previous studies, in line with several pieces of evidence from the literature on human patients, already demonstrated that robotic rehabilitation alone was able to improve forelimb motor function after stroke in mice, but only if we considered the retraction task on the platform. These improvements were not generalized to other forelimb functions [3] indicating that motor rehabilitation alone, even when guided by robotic devices that help to increase the number and the repeatability of the task, is not sufficient to guarantee a true recovery instead of a motor compensation strategy. For these reasons, we implemented combined treatment approaches [1–3] where a plasticizing treatment is applied to prime the spared motor tissue and to make it more responsible for activity-dependent plasticity. Thanks to this approach, we’ve been able to validate several possible rehabilitation strategies to offer to the clinical practice for possible translations on human patients. Here, our hypothesis was that local 40 Hz stimulation, synchronized with forelimb motor training, could effectively engage the surviving PV-INs in perilesional tissue, thereby enhancing movement-related gamma band oscillations and promoting motor recovery. We first combined daily robotic rehabilitation with gamma band stimulation via direct PV-IN activation using optogenetics. Initiating treatment 5 days poststroke, during the subacute phase, allowed us to exploit the plastic critical period and accommodate the physiological delay necessary for engaging in rehabilitative exercises, mandatory for human patients. This clinically relevant approach resulted in significant improvements of forelimb function, with benefits sustained even after a period without intervention. This successful protocol leverages the consistent action potential output of PV-INs evoked by 40 Hz light pulses -which align with the kinetics of the ChR2 channel [20,75–77] to restore forelimb function in motor tests. Notably, robotic rehabilitation alone, or combined with 8 Hz stimulation, did not yield similar improvements, reinforcing our previous findings [1–3]. Indeed, robotic-guided motor rehabilitation alone—despite enhancing task repetition and consistency—is insufficient for true recovery and instead promotes task-specific motor compensation strategies.

Considering these results, we focused on noninvasive neuromodulation techniques—particularly tACS—for their translational potential [78]. These methods have been validated both clinically [79,80] and in animal models [81]. By leveraging noninvasive techniques like tACS, which can entrain specific brain rhythms either at rest or during task performance while requiring minimal current to achieve desired effect [82], this strategy offers accessible and effective rehabilitation solutions for human patients, eliminating the need for invasive procedures such as optogenetics. Moreover, it is well-established that tACS is more effective when applied during tasks aligned with the target frequency of brain activity [83,84]. This makes tACS protocols fit well with the crucial necessity of physical rehabilitation for stroke patients.

We consistently maintained a stimulation frequency of 40 Hz because high-frequency stimulation preferentially synchronizes fast-spiking IN, whereas lower frequencies mainly regular-spiking neurons. It has been already demonstrated that gamma tACS facilitates motor performance in healthy individuals [85–87]. Unfortunately, at present there is no consensus on the optimal tACS protocol for stroke treatment [88]. Here, we demonstrated the feasibility of replicating the positive results obtained via 40 Hz optogenetic stimulation of PV-INs by employing less invasive gamma tACS over the perilesional RFA. This multi-session rehabilitation protocol not only effectively increased gamma power during active movement but also resulted in sustained motor improvement even after a period without rehabilitation.

Interestingly, we revealed that the functional improvements were not associated with a normalization of the prestroke symmetrical network architecture. Instead, motor recovery relied on persistent adaptive FC within the contralesional hemisphere. Despite tACS being delivered over the ipsilesional hemisphere, we did not observe significant changes in intra-hemispheric connectivity within this region. This may be a consequence of the extensive damage affecting local circuits, limiting their plastic rearrangement. Nevertheless, stimulation of the perilesional cortex was sufficient to induce remote effects, selectively reducing hypoconnectivity in the contralesional hemisphere and between hemispheres. These findings highlight how neurostimulation of peri-infarct areas promotes recovery by engaging intact remote circuits, supporting the idea that poststroke functional compensation relies on alternative network recruitment rather than restoration of damaged circuits.

We further examined the morphological and functional changes induced by combined rehabilitation within the perilesional RFA. Our findings indicate that gamma band stimulation significantly influences PV-IN connections, supporting the close relationship between gamma band oscillations and the excitation/inhibition balance [19,20] within networks of GABAergic IN and pyramidal cells in the primary motor cortex [15–17] Notably, gamma oscillations appear to induce homeostatic changes in the inhibitory system, as evidenced by our immunohistochemical analysis showing increased expression of GABAergic transporter following treatment. This suggests enhanced cellular responsiveness to the strengthening of the PV-IN circuit following neurorehabilitation, which may also mitigate further neuronal loss during the acute phase. However, in the chronic phase, the role of GABA becomes more complex. Poststroke alterations in GABA release can considerably alter the expression of GABA-A receptors, thereby modifying both synaptic (phasic) and extrasynaptic (tonic) inhibition. This has led to conflicting findings in literature. Recent studies, such as Clarkson and colleagues [89], have focused on manipulating tonic inhibition, showing that decreasing it after stroke improves motor performances in mice. These results were also confirmed in our laboratory, where modulating presynaptic GABA signaling in the first week after stroke in a mouse model improves long-term motor functions [69]. In contrast, literature reports conflicting effects when modulating phasic inhibition [90], reporting an increase in phasic inhibition in perilesional areas during the critical window of cortical plasticity in a mouse model of stroke. By positively modulating this phasic inhibition with Zolpidem, an improvement in the motor performance of the animals was obtained. More recently, a selective increase of phasic inhibition has been demonstrated after a treatment with “continuous theta bursts simulation” in mice with an ischemic lesion and a corresponding functional improvement. These results suggest a positive role of increased phasic inhibition in the postacute phase of ischemic injury, but this topic is still debated [91].

In summary, our study demonstrates the effectiveness of a novel, clinically valuable, neurorehabilitation approach that combines physical therapy with movement-related gamma band stimulation. Our results highlight that gamma band stimulation can significantly enhance motor function by engaging and strengthening neural circuits within the perilesional and distal cortices. The integration of robotic therapy further enhances these beneficial effects, providing a practical and scalable tool for neurorehabilitation. Given the robust restoration of PV-IN-mediated gamma modulation and the significant motor recovery observed in our mouse model, our findings offer significant proof-of-concept for this integrated approach. Future clinical trials will be critical to confirm its translational potential and facilitate its widespread adoption, ultimately improving the quality of life and long-term outcomes for stroke survivors.

### Limitations of the study

This study raises several important questions and highlights issues that warrant further investigation with different techniques. First, while animal models provide valuable insights, differences in nervous system complexity and brain size between rodents and humans may affect the precision and applicability of tACS treatment in clinical settings. Furthermore, the precise molecular and electrophysiological mechanisms of tACS are not entirely understood and the effect observed at a molecular point of view requires further investigations. Electrical stimulation with tACS protocol has a totally different mechanism of stimulation of brain circuitry with respect to optogenetics, so long as it lacks specificity for a single cell population. From our experiments, we can argue that tACS is able to synchronize brain oscillations on gamma rhythm across all the cortical layers, as reported in Fig 6, even if we cannot report cellular or molecular mechanisms underlying this effect. The evaluation of tACS as a standalone intervention is beyond the scope of this work as our focus was on rehabilitative outcomes and physical rehabilitation is a standard therapy for poststroke patients. Moreover, the photothrombotic lesion method used in this study, although advantageous in terms of reproducibility and spatial specificity, does not perfectly mimic human ischemic pathology due to its lack of a penumbra and highly focal nature. Additionally, because of the relatively slow temporal kinetics of the calcium indicator GCaMP7f, our widefield experiment was not suitable for detecting gamma-band activity. Although PV-INs are critically involved in gamma-band oscillations, the calcium signals we measured do not directly reflect gamma-frequency fluctuations. These limitations underscore the need for further refinement of these methods and their adaptation for clinical use. Nevertheless, the high translatability of the results obtained with tACS -combined with its well-tolerated, noninvasive nature- and the rapidly expanding integration of robotic devices in poststroke rehabilitation strongly support the prompt initiation of clinical trials to validate and potentially implement this integrated rehabilitative approach.

## Materials and methods

### Study design

This study included 49 C57BL6/J and 37 B6;129P2-Pvalb-tm1(cre)Arbr/J (PV::Cre, Jackson Laboratories, JAX stock #017320) adult mice, aged 2–3 months. Animals were housed under standard conditions with a 12 h light/dark cycle and free access to food and water. All experimental procedures respected the ARRIVE guidelines and the European Communities Council Directive #86/609/EEC were approved by the Italian Ministry of Health (protocol #684/2020-PR, integrated on 19/01/2023, 753/2015-PR dated 27/07/2015, 723/2019-PR). Animal numbers were minimized in line with the 3Rs principle, particularly Reduction, to ensure ethical use of animals while maintaining sufficient statistical power. The use of electrophysiological recordings and wide-field calcium imaging in awake, head-fixed mice with chronic implants during behavioral tasks necessitated technically demanding and longitudinal protocols. Accordingly, within-subject experimental designs and repeated acquisition sessions were employed to maximize data yield per animal and reduce inter-individual variability. Sample sizes were informed by prior studies and pilot data to balance scientific validity with ethical considerations and power calculations were performed using G Power Software (v3.1.5). Animals were assigned randomly to groups.

### Ischemic lesion

Cortical ischemic damage in the CFA was induced using the photothrombosis method, as previously reported [3]. Briefly, animals were anesthetized with ketamine/xylazine (100/10 mg/kg i.p.) and placed in a stereotaxic apparatus. After a midline scalp incision, the skull was cleaned and dried. Subsequently, Rose Bengal (0.2 ml 10 mg/ml in phosphate-buffered saline (PBS); Sigma Aldrich) was intraperitoneally injected. After 5 min, the brain was illuminated through the intact skull for 15 min using a cold light source (ZEISS CL 6000, Germany) linked to a 20× objective positioned over the CFA of the right hemisphere (0.5 mm anterior and 1.75 mm lateral from Bregma, using a motorized micromanipulator (Sutter Instruments, USA). Sham animals underwent scalp incision and Rose Bengal injection but were not subjected to light irradiation. Following the photothrombotic procedure, animals underwent a head-restraining implantation surgery, consisting in a metal L-shaped bar posted on the occipital bone using dental cement (Super Bond C&B, Sun Medical Company, Japan). For electrophysiology, a metal screw connected to a ground electrode was implanted in the occipital bone, and a craniotomy was performed to expose the RFA (2.0 mm anterior and 1.25 mm lateral to Bregma [36]). The recording chamber was created by encircling the craniotomy hole with dental cement, then covered with agarose (1% in physiological solution) and silicon (Kwik-Cast Sealant, World Precision Instruments, USA) to protect the underlying tissues. For optogenetics, an optical fiber was positioned over the craniotomy. For tACS, a plastic tube was cemented over the RFA.

#### Time points evaluation.

In mice, the 2–5 day poststroke period is considered an “early subacute phase” that is crucial for the initiation of the rehabilitation treatment. Accordingly, for both electrophysiology and wide-field imaging, data were collected at day 2 and day 5 poststroke, and subsequently once per week during the subacute phase. Additionally, if no plasticizing treatment is applied, 30 days poststroke is considered chronic. Therefore, in the wide-field experiments, the imaging was performed until day 28 (also considering a possible decrease in window clarity and signal quality). Conversely, all treatments were applied for 37 days poststroke, with 1 week of follow-up without treatment; and for consistency, the postrehabilitation wide-field experiment was carried out for 44 days.

Behavioral assessment was conducted before the induction of the stroke (baseline) and tracking the evolution of the lesion from the acute to the chronic phase at 2, 9, 16, 23, 30, and 37 days postlesion.

### Viral injection

PV::Cre transgenic mice received a stereotactic injection of the AAV vector (AAV1.EF1.dflox.hChR2(H134R)-mCherry.WPRE.hGH (Addgene, USA) to induce a Cre-dependent expression of Channelrhodopsin-2 (ChR2). Briefly, a craniotomy was performed in RFA and 600 µl of AAV was injected in RFA 800 µm below the dura, with a flow rate of 0.1 µl/min with a Legato 130 syringe pump (kdScientific) and a 10 µl Hamilton syringe (Hamilton Company). PV-Cre mice express Cre recombinase in parvalbumin-expressing neurons. The term PV-ChR2 denotes PV-Cre mice injected with the AAV. The surgery was performed under a cocktail of ketamine/xylazine (100/10 mg/kg i.p.). For PV-INs labeling with GCaMP7f, the viral construct ssAAV-PHP.eB/2-hSyn1-chl-dlox-jGCaMPf(rev)-dlox-WPRE-SV40p(A) (Viral Vector Facility, CH) was intravenously injected in the retro-orbital sinus of PV-Cre mice under isoflurane anesthesia. A representative image of GCaMP7f expressing cells with quantification of viral efficiency and specificity is shown in S2G and S2H Fig.

### Wide-field calcium imaging

#### WF setup.

Imaging was performed through the intact skull using a custom-made microscope. The microscope consisted of back-to-back 50 mm f/1.2 camera lenses (Nikon). To excite the GCaMP7f indicator, a 470 nm light source (LED, M470L3, Thorlabs) filtered by a bandpass filter (469/17.5 nm, Thorlabs) was deflected by a dichroic filter (MD498, Thorlabs) on the objective (TL2X-SAP 2× Super Apochromatic Microscope Objective, 0.1NA, 56.3 mm WD, Thorlabs). Reflectance images were acquired using a light source positioned at 45° incident to the brain surface (530 nm LED light, M530L4; Thorlabs, New Jersey, USA). Stroboscopic illumination (25 Hz/LED) was used. The fluorescence and reflectance signals were selected by a bandpass filter (525 ± 19.5 nm Thorlabs) and collected by a CMOS camera (Orca 4.0 v2, Hamamatsu). Images were acquired at 50 Hz, with a resolution of 512 × 512 pixels with a FOV of 11.5 × 11.5 mm.

#### Surgery.

One week after AAV injection, mice were implanted with an intact skull preparation to allow free optical access to the cortex (modified from [2,92]). The skin and the periosteum were removed. Bregma was marked for stereotactic reference. A custom-made aluminum head-bar placed behind lambda was glued to the skull using transparent dental cement (Super Bond C&B—Sun Medical). The exposed cortex was then covered with the same cement. One week after the surgery, mice were habituated to head fixation under the wide-field microscope before the first imaging session. Calcium imaging was performed on awake, head-restrained PV-CRE mice during resting state the week before and 5–30 days after stroke. Each animal was imaged for approximately 30 min, including head fixation, five consecutive imaging blocks of 120 s, data storage between blocks, and head-fixation release.

#### Habituation and imaging protocol.

Following recovery from surgery for at least 1 week, mice were habituated to the experimental setup for 3–4 d (20 min a day/mouse) to gradually reduce anxiety and abrupt movements before imaging sessions. Then, mice were gently placed under the microscope objective and fixed. Cortical activity was recorded in awake, head-restrained mice that could freely move their limbs and weren’t engaged in any distinct tasks. Each session contained at least 5 blocks, and each block lasted 120 s.

#### Image processing and data analysis.

Image stacks for each animal collected from different sessions were registered using custom-made software, by taking into account the bregma and λ position. An animal-specific field of view template was used to manually adjust the imaging field daily. To dissect the contribution of each cortical area, we registered the cortex to the surface of the Allen Institute Mouse Brain Atlas (www.brain-map.org) projected to our plane of imaging. For each block, image stacks were processed to obtain the estimates of Δ*F*/*F*0. Δ*F*/*F* was computed for each pixel, where Δ*F* was the intensity value of that pixel in a specific time point and *F* was the mean fluorescence intensity of the signal across time. Hemodynamic correction was performed as described by Scott and colleagues [93]. Briefly, using the ratiometric approach:


$$\frac{F}{F_{\text{0}}}=\frac{\frac{I^{\text{4}\text{8}\text{2}}}{I_{\text{0}}^{\text{4}\text{8}\text{2}}}}{\frac{I^{\text{5}\text{2}\text{5}}}{I_{\text{0}}^{\text{5}\text{2}\text{5}}}}$$

where *F*/*F*_0_ is the final corrected GCaMP7f time series for a given pixel, *I*^482^ refers to the detected fluorescence signal, *I*^525^ is the reflectance signal. Then, GSR was applied.

“Inter-hem” refers to inter-hemispheric FC. “Intra-hem” refers to intra-hemispheric FC. “ipsi” refers to the ipsilateral hemisphere while “contra” refers to the contralateral hemisphere. Whole cortex connection strength was calculated by averaging all the values within the correlation matrix. Calculations of intra-hemispheric FC changes across time were performed by averaging correlation values (Fisher z-transformed) within all the left hemisphere (contralateral) or all the right hemisphere (ipsilateral). Inter-hemispheric FC was obtained by averaging correlation values between all right- and left-hemisphere region pairs. Homotopic FC was obtained by averaging correlation values between the same brain regions in the opposite hemisphere.

A total of 22 ROIs were then selected (11 ROI for each hemisphere, 20 × 20 pixels). Correlation mapping was done for each subject by computing Pearson’s correlation coefficient between the average signals extracted from each ROI, with that of each other ROI. The single-subject correlation maps were then transformed using Fisher’s r-to-z transform and then averaged across all animals. Averaged maps were re-transformed to correlation values (r-scores). For each mouse, r(prestroke) − r(x-day poststroke) was calculated and averaged across mice in order to visualize matrices of difference between the prestroke condition and all the time points after-stroke. No temporal filtering was applied prior to FC analysis.

### Forelimb retraction task on the M-Platform

The M-Platform, a robotic device for functional assessment and for neurorehabilitation of the forelimb in mice [1,27], consists of a linear actuator (Micro Cylinder RCL, IAI, Germany), a load cell (Nano 17, ATI Industrial Automation, USA) with a controlled friction system and a customized handle placed on a precision linear IKO slide (IKO BWU 25-75, USA) fastened to the left wrist of the animal. The handle is attached to the load-cell for loss-less transfer of the forces to the sensor, while providing support for the animal’s wrist. The animal is kept in a U-shaped restrainer, and its head is fixed throughout the cemented post. Daily training sessions were conducted for both electrophysiological recordings and rehabilitation experiments. Each session involved 15 forelimb retractions by each mouse, combining passive (device-extended by 10 mm) and active (animal-retracted) movements. Overcoming a force threshold earned the mice a liquid reward for each successful task completion. Mice typically mastered this task, improving performance within 2–3 days [27]. Rehabilitation began 5 days postlesion and continued until day 37, occurring for 4 consecutive days a week. Adjustments to task friction were made according to each animal’s functional deficit. Details on coupled sham or neuromodulatory treatments are provided below.

### Optogenetic stimulation

Optogenetic stimulation was employed for identifying PV-INs during electrophysiological recordings and as a neuromodulatory treatment for rehabilitation. The stimulation setup included a PlexBright Optogenetic Stimulation System with an LD-1 Single Channel LED Driver and a 456 nm LED Module (PlexonInc, USA), connected to a 200 µm Core 0.39 NA optic fiber (ThorlabsInc, USA). Before each experiment, the maximum emission power of the optic fiber was measured using the PlexBright Light Measurement Kit (~10, 79.55 mW/mm^2^). Based on a safe range of ~75 mW/mm^2^ for short pulses (0.5–50 ms) [94], we limited optogenetic stimulation to 31.82 mW/mm^2^ (40% of maximum). We can assume that the blue light with our stimulation parameter can actually reach the lower layers based on calculations of the actual irradiance of the optic fiber through organic tissue already performed with specific simulators (https://web.stanford.edu/group/dlab/cgi-bin/graph/chart.php). Control of stimulation parameters was managed via custom software in LabWindows/CVI, interfacing through a NI USB-6212BNC DAQ board (National Instruments, USA). For optogenetic identification of PV-INs, we delivered single 200-ms light pulses [95] at 0.2 Hz with increasing light power to ensure that the recorded activity was due to stimulation rather than the cell’s intrinsic activity. For gamma band induction, more physiological 1 ms light pulses were administered (at either 40 or 8 Hz) continuously throughout the task—from the passive to retraction phases on the M-Platform—with task duration varying between 5 and 8 min depending on each animal’s performance.

### Electrophysiological recording

In C57Bl6/J and PV-ChR2 mice, gamma power modulation and PV-INs discharge properties in the RFA were assessed using 16-channel linear probes (NeuroNexus, USA). Mice were head-fixed on the M-Platform, with the left forepaw linked to a load cell, and electrodes were stereotactically inserted in the right hemisphere’s RFA at a depth of 850 µm.

Neural signals were acquired and amplified using DigiAmp (Plexon, USA), with ground in the cerebellum. Recordings were made during resting and retraction task. Offline analysis, performed with custom algorithms in NeuroExplorer (Plexon, USA) and Matlab, involved computing power spectral density (PSD) and synchronizing neural signals with the force signal from the platform. Isolated force peaks (force peaks with no movement within 2.5 s before and after the movement onset) were selected. Peri-events spectrogram analysis was used to calculate the power within the 31−49 Hz frequency band in 0.5-s intervals at three different time windows relative to the onset of the force peak: Baseline (−2 to −1.5 s), Preonset (−0.5 to 0 s), and Postonset (0 to 0.5 s). Ratios of Preonset/Baseline and Postonset/Baseline were computed to evaluate gamma band power variations in CFA during movement with respect to the No-Movement state. Gamma power variations were quantified as percentages of the total PSD, excluding the force peaks and the time windows immediately before and after the movement onset.

Optogenetic identification of PV-INs during resting states involved light pulses administered near the recording electrode. After the optogenetic stimulation protocol, neural activity was recorded during the retraction task without moving the recording electrode. To identify putative PV-INs, whose firing rate increased selectively during optogenetic stimulation. Spike Sorting analysis for single units was performed offline using Offline Sorter Software (Plexon, USA) and was used to refine the principal component analysis (PCA) to exclude potential spikes originating from neighboring neurons. Once identified, putative PV-INs peri-event rate histogram was referred to the movement onset to verify firing rate alignment with voluntary movement. Recordings spanned 3 days to enhance identification of PV-INs involved in motor tasks.

### Behavioral motor tests

Functional assessment of forelimb motor performances was used to evaluate the impact of the ischemic damage and the applied neurorehabilitative protocols. Mice performances were assessed in baseline condition and then once a week at days 2, 9, 16, 23, 30, 37, and 44 postlesion, as reported in Lai and colleagues [96] Two behavioral tests have been used, Gridwalk and Schallert Cylinder test.

In the *Gridwalk test*, mice were allowed to move freely on an elevated grid (32 × 20 cm, with 11 × 11 mm large openings, Micromecc, Italy) for 5 min and the task was video recorded (SMXF50BP/EDC, Samsung, Seoul, South Korea) by a camera positioned in front of the Gridwalk apparatus. Off-line analysis of the videos involved a custom-designed Graphical User Interface implemented in Matlab [96], to assess correct steps and foot-faults, namely, steps not providing body support, with the foot falling into a grid hole. The percentage of foot faults for each limb was then calculated.

In the *Schallert Cylinder test*, animals were placed in a custom-made Plexiglas cylinder (8 cm diameter, 15 cm height) recorded for 5 min by a video-camera (SMXF50BP/EDC, Samsung, Seoul, South Korea) placed below the cylinder. Videos were analyzed frame by frame and the spontaneous use of both forelimbs was assessed during exploration of the walls, by counting the number of contacts performed by the paws of the animal. For each wall exploration, the last paw that left and the first paw that contacted the wall or the ground were assessed. In order to quantify forelimb-use, percentage of contralesional forelimb contacts over the total number of single-paw contacts was calculated as % of Contralateral Forelimb = (C_contra/C_contra + C_ipsi) * 100 where C_ipsi and C_contra correspond to the number of touching performed with the limb ipsilateral and contralateral to the lesioned hemisphere when the mouse was leaning at the vertical walls.

The experimenters were blind to all the experimental groups in both motor tests.

### Transcranial Alternating Current Stimulation (tACS)

tACS was administered using AnimaltES Model 2101 (Soterix Medical, USA) at a frequency of 40 Hz. The cathode was positioned in a saline-filled tube cemented to the skull, and the anode was placed under the abdomen against a saline-soaked sponge. Stimulation began 5 min prior to task onset, with current gradually increasing to a maximum of 0.2 mA, remaining below the movement threshold for mice. The stimulation continued for the task duration (5–8 min), with current ramp-down starting 10 min posttask initiation. Sham stimulation involved electrode placement without current flow. No signs of discomfort or freezing were observed in the animals during tACS.

### Histology

Mice underwent anesthesia with Chloral hydrate followed by cardiac perfusion using 0.01 M PBS (Sigma Aldrich) and 4% paraformaldehyde (PFA, Electron Microscopy Sciences) in 0.1 M Phosphate Buffer. Brains were postfixed with 4% PFA for 2 h, rinsed with 30% sucrose (Sigma Aldrich) in Phosphate Buffer at 4 °C, and sectioned coronally using a sliding microtome (Leica, Germany) to obtain 50 µm thick slices maintained free-floating in PBS for additional treating.

For immunostaining, brain slices were incubated in a blocking solution for 1 h (10% donkey serum; 0.3% Triton X-100 in PBS) and treated with primary antibodies overnight at 4 °C. As primary antibodies, Guinea Pig a-Parva (1:300, SynapticSystem) was used for PV-INs staining and a-VGAT (1:1,000, Synaptic System), for staining vesicular transporters of GABA and Glutamate, respectively. Following three washes in PBS, the sections were incubated for 2 h at room temperature with the specific secondary antibodies; therefore, a-GP-Alexa488 (1:200, Synaptic System) for the PV, a-GP-RRX (1:400, Synaptic System) for labeling the transporters of GABA and Glutamate.

The VGAT and PV signals were acquired by airyscan confocal microscope (Zeiss, Germany) with 63× objective and 1.3 digital zoom, in the medial-superficial/deep and lateral-superficial/ deep regions of the peri-wound tissue. Three fields (77 μm × 77 μm) were acquired for each location (three sections per animal). The acquired images were processed using ImageJ (National Institutes of Health, USA) software to analyze the mean fluorescence in puncta-rings around cell bodies of VGAT and nonPV positive neurons. To minimize the variations due to the different quality of immunostaining in the individual mice/sections, the fluorescence in the periwound areas was normalized to values calculated in three reference fields taken in the basal cortices of each coronal section analyzed.

To quantify the lesion volume, one out of every six sections was stained with Hoechst 33258 (Sigma-Aldrich, USA). The ischemic region was imaged with Apotome fluorescence microscopy (Zeiss, Germany) with a 10× objective and its area measured using ImageJ (National Institutes of Health, USA) software. The lesion volume for each animal was calculated by summing up all damaged areas and multiplying the number by section thickness and by 6 (the spacing factor). A total infarction volume in mm^3^ is given as the mean ± standard error of all analyzed animals.

### Statistical considerations

Statistical analyses were conducted on raw data using SigmaPlot 11.0 (Systat Software, USA), Matlab (R2019a), and OriginLab (2018), with a significance threshold set at alpha = 0.05. For behavioral tests (Gridwalk and Schallert Cylinder), two-way Repeated Measures ANOVA with Tukey posthoc tests were applied. Group comparisons utilized one- or two-way ANOVA, depending on the data structure, followed by either Dunnett’s or Tukey’s posthoc tests. *T* tests were used for immunohistochemical analyses and firing rate comparisons. Group-level ROI-based FC differences pre- and poststroke were analyzed using one-way repeated measure ANOVA with Tukey correction. Network-Based Statistic (NBS) Toolbox in MATLAB assessed functional network connectivity [29,30]. Significance was determined at *p* < 0.05, and errors are expressed as Standard Error of Means (SEM), with significance markers (* *P* < 0.05, ** *P* < 0.01, *** *P* < 0.001).

### Data visualization

Data visualization was performed using OriginPro, while figure editing was performed with Affinity Designer 2 (Serif (Europe), Version 2.6.3).

## Supporting information

S1 Fig Referred to Fig 1. **A**, motor performance assessment on the Gridwalk test of sham (gray bar plots, *n* = 9) and stroke (light blue bar plots, *n* = 8) mice before (baseline) and 2 days (D2) after the induction of the lesion. (Two-way RM ANOVA followed by Holm–Sidak Test, *** = *P* < 0.001.) **B**, Quantification of gamma power modulation in sham (gray bar plots) and stroke (blue bar plots) mice 2 days after the induction of the lesion. (Two-way RM ANOVA followed by Holm–Sidak test.) **C** and **D**, Quantification of gamma band power across all the 16 channels spanning all the cortical layers (channel 1 ≃ 50 μm, channel 16 ≃ 800 μm) in pre- and postonset windows, respectively. Healthy mice (Sham, gray, *n* = 9), animals recorded 2 days after stroke (D2, light blue, *n* = 6), mice recorded 5 days after stroke (D5, dark blue, *n* = 8). Two-way ANOVA followed by Tukey test, * *P* < 0.05, ** *P* < 0.01, *** *P* < 0.001. Each dot represents a single animal. Data are shown as mean ± SEM. The data underlying this figure can be found in https://data.mendeley.com/datasets/mw82tzp4rx/1.
(TIFF)

S2 Fig Referred to Fig 2. **A**, The averaged difference correlation matrices, produced by subtracting the poststroke FC (2, 5, 8, 14, or 30 days after injury) from prestroke FC. Red squares indicate poststroke hypo-connectivity, blue squares indicate poststroke hyper-connectivity of PV-IN. **B**, Averaged correlation matrices before (left) and 2 days after stroke (right) without hemodynamic correction. **C**, The averaged difference correlation matrices, for the Robot tACS group. **D**, Box charts showing inter-hemispheric FC of ipsilesional and contralesional secondary and primary motor cortices in the anterior regions (MOsa and MOpa, respectively) for the Robot tACS group. **E**, Intra-hemispheric FC of contralesional areas for the Robot tACS group. **F**, The box charts displaying intra-hemispheric FC of the secondary (left) and primary (right) contralesional motor cortices in the anterior region for the Robot tACS group. **G**, Representative image of GCaMP7f expressing cells (in green), PV-INs (in red), in a section of coronal brain slice; on the left a magnification of the blue highlighted area is provided for single channel and merge (300 × 1,000 μm). Scale bar: 500 μm. H, Top, quantification of the viral efficiency (90.61 ± 2.56%) and, bottom, specificity (77.72 ± 3.97%) of viral infection in the PV-Cre mouse model (*n* = 4). I average lesion volume at 30 days poststroke without any treatment (white, *n* = 65) and in Robot + tACS (red, *n* = 6) (*t* Test, *p* = 0.57). **J**, Motor performance assessment with the gridwalk test. Animals displayed a significant level of motor impairment at D2 postlesion while at follow-up (FU) the performances were comparable to baseline. (One-way RM ANOVA followed by Tukey test, ** = *P* < 0.01, *** = *P* < 0.001.) Each dot represents a single animal. Data are shown as mean ± SEM. The data underlying this figure can be found in https://data.mendeley.com/datasets/mw82tzp4rx/1.
(TIF)

S3 Fig Referred to Fig 5. **A**, Quantification of lesion volume in stroke-affected animals that underwent no intervention (No treatment, dark gray, *n* = 6), robotic rehabilitation alone (Robot, light gray, *n* = 6) and robotic rehabilitation in combination with tACS (Robot + tACS, red, *n* = 6). No significant differences were observed in the lesion volume between the three groups. **B**, Motor performance assessment with the gridwalk test. Animals displayed a significant level of motor impairment at D2 postlesion. However, only the robot-tACS group showed performance comparable to baseline during the follow-up period (FU). (Two-way RM ANOVA followed by Tukey test, * = *P* < 0.05, ** = *P* < 0.01, *** = *P* < 0.001.) **C** and **D**, Quantification of gamma band power across all the 16 channels spanning all the cortical layers (channel 1 ≃ 50 μm, channel 16 ≃ 800 μm) in pre- and postonset windows, respectively. Mice treated with rehabilitation alone (gray bar plots, n = 7) and coupled with noninvasive 40 Hz tACS (red bar plots, *n* = 6). Two-way ANOVA followed by Tukey test, * *P* < 0.05, *** *P* < 0.001. Each dot represents a single animal. Data are shown as mean ± SEM. The data underlying this figure can be found in https://data.mendeley.com/datasets/mw82tzp4rx/1.
(TIFF)

S4 Fig Referred to Fig 2. **A**, Pairwise Pearson’s correlation coefficients of cortical activity for each imaging time point in the stroke group after global signal regression. **B**, Network diagrams of statistically significant FC alterations after 2, 5, 8, 14, 21, or 28 days from injury. Blue and red lines denote significant hyper-correlation and hypo-correlation compared to prestroke values, respectively. The bar plots (bottom) indicate the number of significant FC alterations for each cortical area. **C**, Box chart illustrating FC averaged over the whole cortex. **D**, Box chart illustrating the averaged inter-hemispheric FC. **E**, Box charts showing inter-hemispheric FC of ipsilesional and contralesional secondary and primary motor cortices in the anterior regions (MOsa and MOpa respectively). **F**, Intra-hemispheric FC of ipsi-lesional areas. **G**, Box charts displaying intra-hemispheric FC of the secondary (left) and primary (right) ipsilesional motor cortices in the anterior region. **H**, Intra-hemispheric FC of contralesional hemisphere. **I**, the box charts display intra-hemispheric FC of the secondary (left) and primary (right) contralesional motor cortices in the anterior region. **J**, Homotopic FC changes from prestroke to 28 days after injury (MOsa, anterior secondary motor cortex; MOsp, anterior primary motor cortex; SSp.tr, primary somatosensory cortex-trunk; RSP, dorsal part of the retrosplenial cortex; VISp, primary visual cortex). One-way ANOVA followed by Tukey test, * *P* < 0.05, ** *P* < 0.01, *** *P* < 0.001. Data are shown as mean ± SEM. Each color indicates a single subject, *n* = 5. The data underlying this figure can be found in https://data.mendeley.com/datasets/mw82tzp4rx/1.
(TIF)

S5 Fig Referred to Fig 6. **A**, Pairwise Pearson’s correlation coefficients of cortical activity for each imaging time point in the Stroke + tACS group after global signal regression. **B**, Network diagrams of statistically significant FC alterations after 2, 5, 8, 14, 21, or 28 days from injury. Blue and red lines denote significant hyper-correlation and hypo-correlation compared to prestroke values, respectively. The bar plots (bottom) indicate the number of significant FC alterations for each cortical area. **C**, Box chart illustrating FC averaged over the whole cortex. **D**, Box chart illustrating the averaged inter-hemispheric FC. **E**, Intra-hemispheric FC of ipsi-lesional areas. ****F,** **Box charts displaying intra-hemispheric FC of the secondary (left) and primary (right) ipsilesional motor cortices in the anterior region. **G**, Homotopic FC changes from prestroke to 28 days after injury (MOsa, anterior secondary motor cortex; MOsp, anterior primary motor cortex; SSp.tr, primary somatosensory cortex-trunk; RSP, dorsal part of the retrosplenial cortex; VISp, primary visual cortex). One-way ANOVA followed by Tukey test, * *P* < 0.05, ** *P* < 0.01, *** *P* < 0.001. Data are shown as mean ± SEM. Each color indicates a single subject, *n* = 6. H-K Comparison of changes in functional connectivity (ΔFC) relative to baseline (prestroke) between Stroke (gray) and Robot + tACS (dark red) groups across different time points after stroke (D2–D28) in terms of whole-cortex (**H**), inter-hemispheric connectivity (**I**), intra-hemispheric connectivity of ipsilesional hemisphere (**J**), and intra-hemispheric connectivity of the contralesional hemisphere (**K**). Data are shown as box plots (mean ± SE). *p < 0.05, main effect of treatment (two sample *t* test). Blue and red arrows indicate the directions of hyper- and hypo-connectivity compared to the prestroke FC, respectively (stroke *n* = 5 mice, robot + tACS *n* = 6 mice). The data underlying this figure can be found in https://data.mendeley.com/datasets/mw82tzp4rx/1.
(TIF)

S6 Fig Referred to Fig 6. **A**, the averaged difference correlation matrices, produced by subtracting the poststroke FC (2, 5, 8, 14, or 30 days after injury) from prestroke FC in the stroke group after global signal regression. Red squares indicate poststroke hypo-connectivity, blue squares indicate poststroke hyper-connectivity of PV-IN. **B**, the averaged difference correlation matrices, produced by subtracting the poststroke FC (2, 5, 8, 14, or 30 days after injury) from prestroke FC in the Robot + tACS group after global signal regression. Red squares indicate poststroke hypo-connectivity, blue squares indicate poststroke hyper-connectivity of PV-IN. **C**, Box charts showing inter-hemispheric FC of ipsilesional and contralesional secondary and primary motor cortices in the anterior regions (MOsa and MOpa, respectively) for the Robot tACS group. **D**, Intra-hemispheric FC of contralesional areas for the Robot tACS group. **E**, The box charts displaying intra-hemispheric FC of the secondary (left) and primary (right) contralesional motor cortices in the anterior region for the Robot tACS group. One-way ANOVA followed by Tukey test, * *P* < 0.05, ** *P* < 0.01, *** *P* < 0.001. Data are shown as mean ± SEM. Each color indicates a single subject, *n* = 6. The data underlying this figure can be found in https://data.mendeley.com/datasets/mw82tzp4rx/1.
(TIF)

S7 Fig Referred to Fig 2. **A**, Power spectral density (PSD) of GCaMP7f fluorescence and 530 nm reflectance signals from left CFA region (in red) and left SSp-bfd region (in blue) in wake resting-state condition prestroke (ROI: 5 × 5 pixels, FOV: 128 × 128 pixels). These regions are clearly indicated in the inset of the figure (scale bar: 1 mm, white dot indicates bregma). For each region, we report the raw (uncorrected) GCaMP7f signal (darker color), the signal after correction for hemodynamic contamination (palet tone), and the corresponding 530 nm reflectance trace (lighter color). Data are plotted on a log–log scale to highlight frequency-dependent power and the presence of 1/*f* dynamics (*n* = 1). **B**, The upper plot shows representative time courses of raw GCaMP7f fluorescence (Δ*F*/*F*, dark red) and 530 nm reflectance (Δ*I*/*I*, light red) signals extracted from the left CFA region (CFA L) during a 120-s widefield optical imaging (WFOI) session. Corrected GCaMP7f fluorescence (bright red) was obtained by regressing out the 530 nm reflectance signal from the raw GCaMP7f signal, to reduce contamination from hemoglobin absorption. The bottom panel presents a magnified view of the time window highlighted in green in the upper plot (10–30 s), allowing clearer visualization of the temporal relationships between the raw and corrected GCaMP7f signals and the hemodynamic component. **C**, The upper plot shows representative time courses of raw GCaMP7f fluorescence (Δ*F*/*F*, dark red) and 530 nm reflectance (Δ*I*/*I*, light red) signals extracted from the left barrelfield region (SSp-bfd L) during a 120-s widefield optical imaging (WFOI) session. Corrected GCaMP7f fluorescence (bright red) was obtained by regressing out the 530 nm reflectance signal from the raw GCaMP7f signal, to reduce contamination from hemoglobin absorption. The bottom panel presents a magnified view of the time window highlighted in green in the upper plot (approximately 10–30 s), allowing clearer visualization of the temporal relationships between the raw and corrected GCaMP7f signals and the hemodynamic component. **D**, Functional connectivity matrices derived from hemodynamic signals before (PRE) and at multiple time points after stroke (2, 5, 8, 14, 21, and 28 days poststroke). Color scale represents Pearson’s correlation coefficient (*r*), ranging from −0.6 to 1 for hemodynamic data (*n* = 5 mice). The data underlying this figure can be found in https://data.mendeley.com/datasets/mw82tzp4rx/1.
(TIF)

S8 Fig Referred to Fig 6. Representative PV-Cre::GCaMP7f resting state cortical activity maps in a mouse at baseline (**A**) and after 2 (**B**), 5 (**C**), 8 (**D**), 14 (**E**), 21 (**F**), 28 (**G**), 36 (**H**), and 43 (**I**) days after stroke. From day 5 to day 28, the mouse received daily tACS. L: lateral; M: medial; R: rostral; C: caudal. Scale bar: 1 mm. The data underlying this figure can be found in https://data.mendeley.com/datasets/mw82tzp4rx/1.
(TIF)

## Abbreviations

CFA: Caudal Forelimb Area
FC: functional connectivity
GSR: global signal regression
PBS: phosphate-buffered saline
PFA: paraformaldehyde
PSD: power spectral density
PV-INs: parvalbumin-positive interneurons
RFA: Rostral Forelimb Area
SEM: Standard Error of Means
tACS: transcranial Alternating Current Stimulation
