# Supplementary figures and images for: Combining gamma neuromodulation and robotic rehabilitation after a stroke restores parvalbumin interneuron dynamics and improves motor recovery in mice
Source: PLoS Biol. 2025 Oct 14;23(10):e3002806. doi: 10.1371/journal.pbio.3002806 (PMC12520394; doi:10.1371/journal.pbio.3002806)

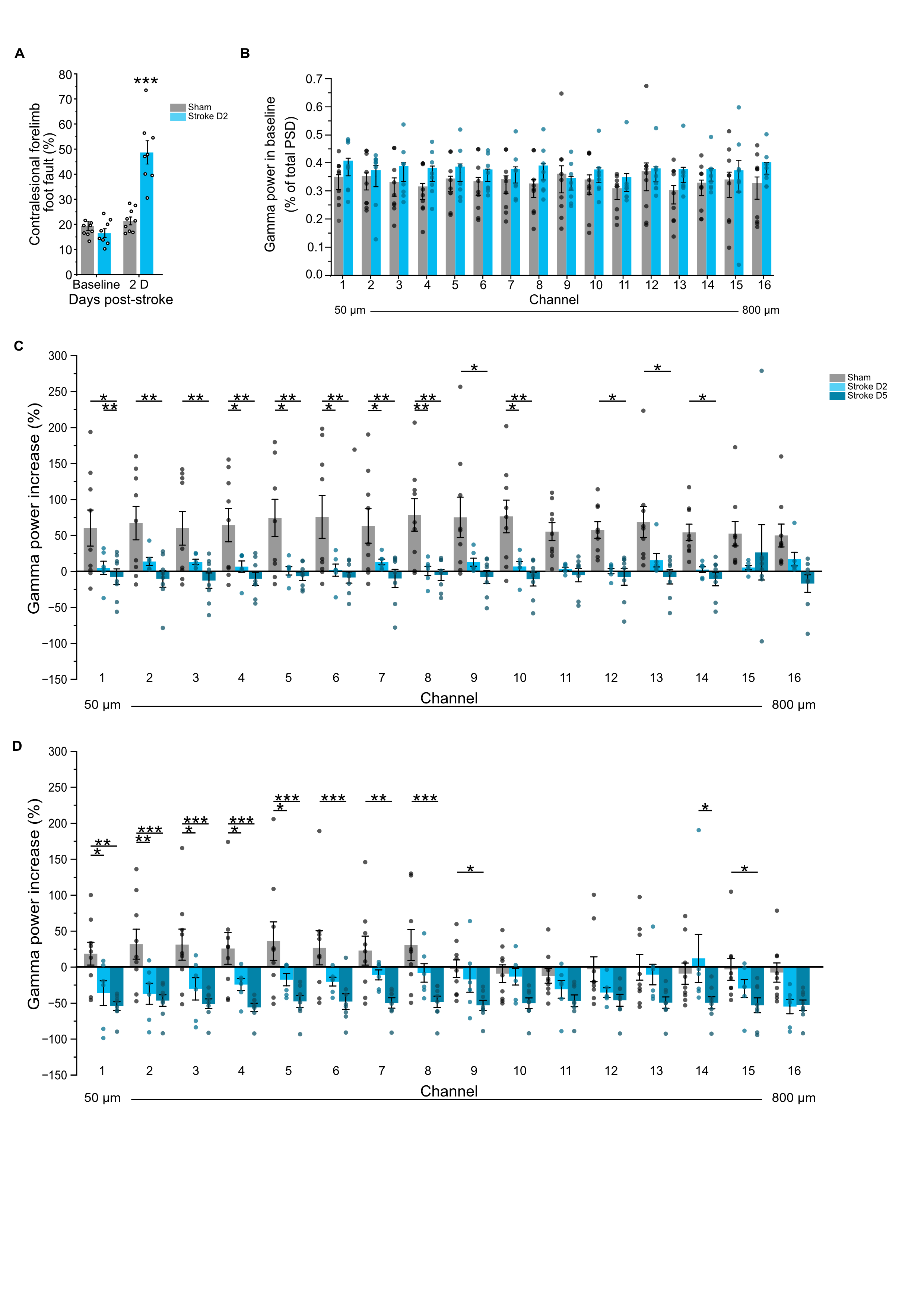

Supplement: S1 Fig — A, motor performance assessment on the Gridwalk test of sham (gray bar plots, n = 9) and stroke (light blue bar plots, n = 8) mice before (baseline) and 2 days (D2) after the induction of the lesion. (Two-way RM ANOVA followed by Holm–Sidak Test, *** = P < 0.001.) B, Quantification of gamma power modulation in sham (gray bar plots) and stroke (blue bar plots) mice 2 days after the induction of the lesion. (Two-way RM ANOVA followed by Holm–Sidak test.) C and D, Quantification of gamma band power across all the 16 channels spanning all the cortical layers (channel 1 ≃ 50 μm, channel 16 ≃ 800 μm) in pre- and postonset windows, respectively. Healthy mice (Sham, gray, n = 9), animals recorded 2 days after stroke (D2, light blue, n = 6), mice recorded 5 days after stroke (D5, dark blue, n = 8). Two-way ANOVA followed by Tukey test, * P < 0.05, ** P < 0.01, *** P < 0.001. Each dot represents a single animal. Data are shown as mean ± SEM. The data underlying this figure can be found in https://data.mendeley.com/datasets/mw82tzp4rx/1. (TIFF) [file pbio.3002806.s001.tiff]

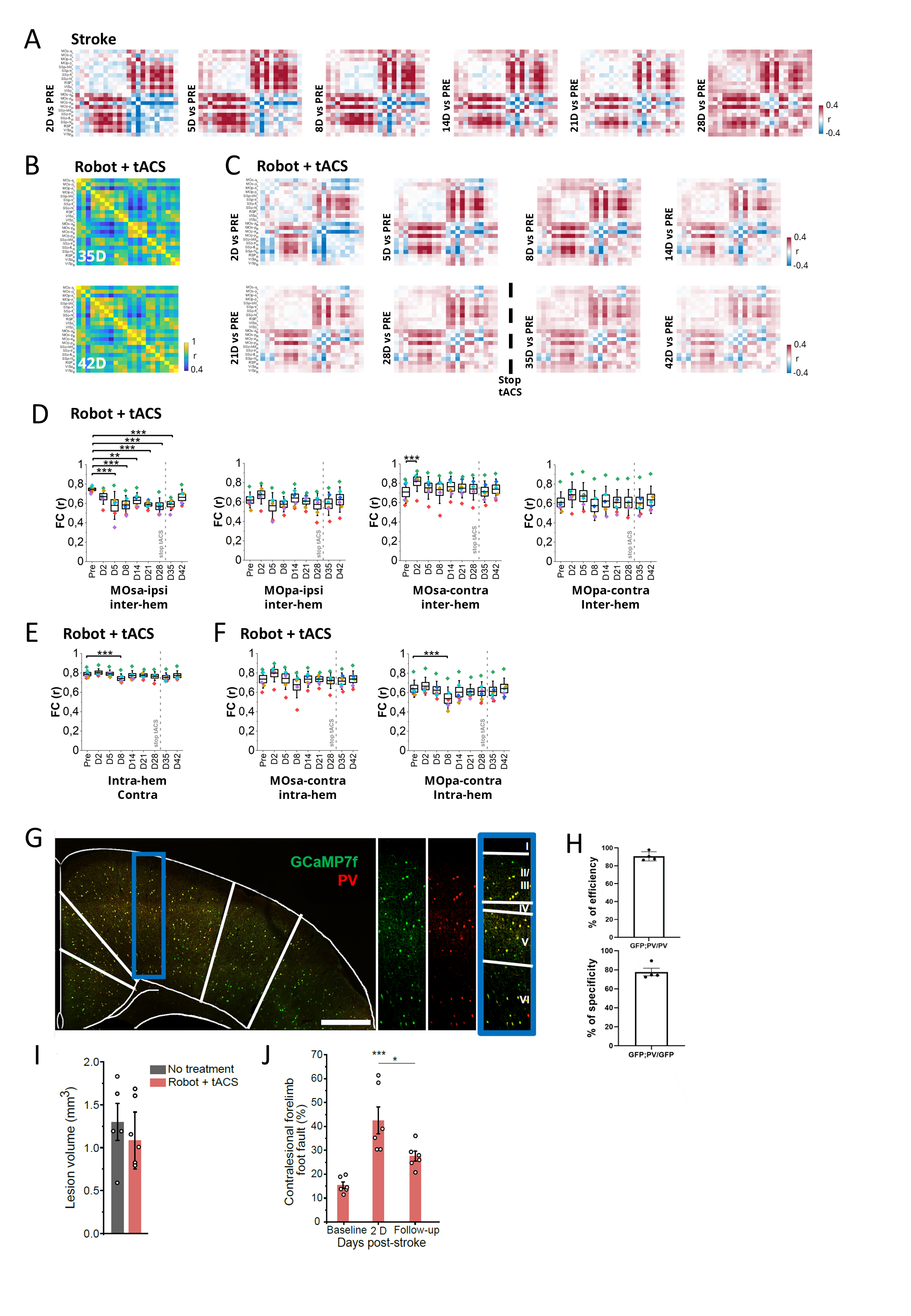

Supplement: S2 Fig — A, The averaged difference correlation matrices, produced by subtracting the poststroke FC (2, 5, 8, 14, or 30 days after injury) from prestroke FC. Red squares indicate poststroke hypo-connectivity, blue squares indicate poststroke hyper-connectivity of PV-IN. B, Averaged correlation matrices before (left) and 2 days after stroke (right) without hemodynamic correction. C, The averaged difference correlation matrices, for the Robot tACS group. D, Box charts showing inter-hemispheric FC of ipsilesional and contralesional secondary and primary motor cortices in the anterior regions (MOsa and MOpa, respectively) for the Robot tACS group. E, Intra-hemispheric FC of contralesional areas for the Robot tACS group. F, The box charts displaying intra-hemispheric FC of the secondary (left) and primary (right) contralesional motor cortices in the anterior region for the Robot tACS group. G, Representative image of GCaMP7f expressing cells (in green), PV-INs (in red), in a section of coronal brain slice; on the left a magnification of the blue highlighted area is provided for single channel and merge (300 × 1,000 μm). Scale bar: 500 μm. H, Top, quantification of the viral efficiency (90.61 ± 2.56%) and, bottom, specificity (77.72 ± 3.97%) of viral infection in the PV-Cre mouse model (n = 4). I average lesion volume at 30 days poststroke without any treatment (white, n = 65) and in Robot + tACS (red, n = 6) (t Test, p = 0.57). J, Motor performance assessment with the gridwalk test. Animals displayed a significant level of motor impairment at D2 postlesion while at follow-up (FU) the performances were comparable to baseline. (One-way RM ANOVA followed by Tukey test, ** = P < 0.01, *** = P < 0.001.) Each dot represents a single animal. Data are shown as mean ± SEM. The data underlying this figure can be found in https://data.mendeley.com/datasets/mw82tzp4rx/1. (TIF) [file pbio.3002806.s002.tif]

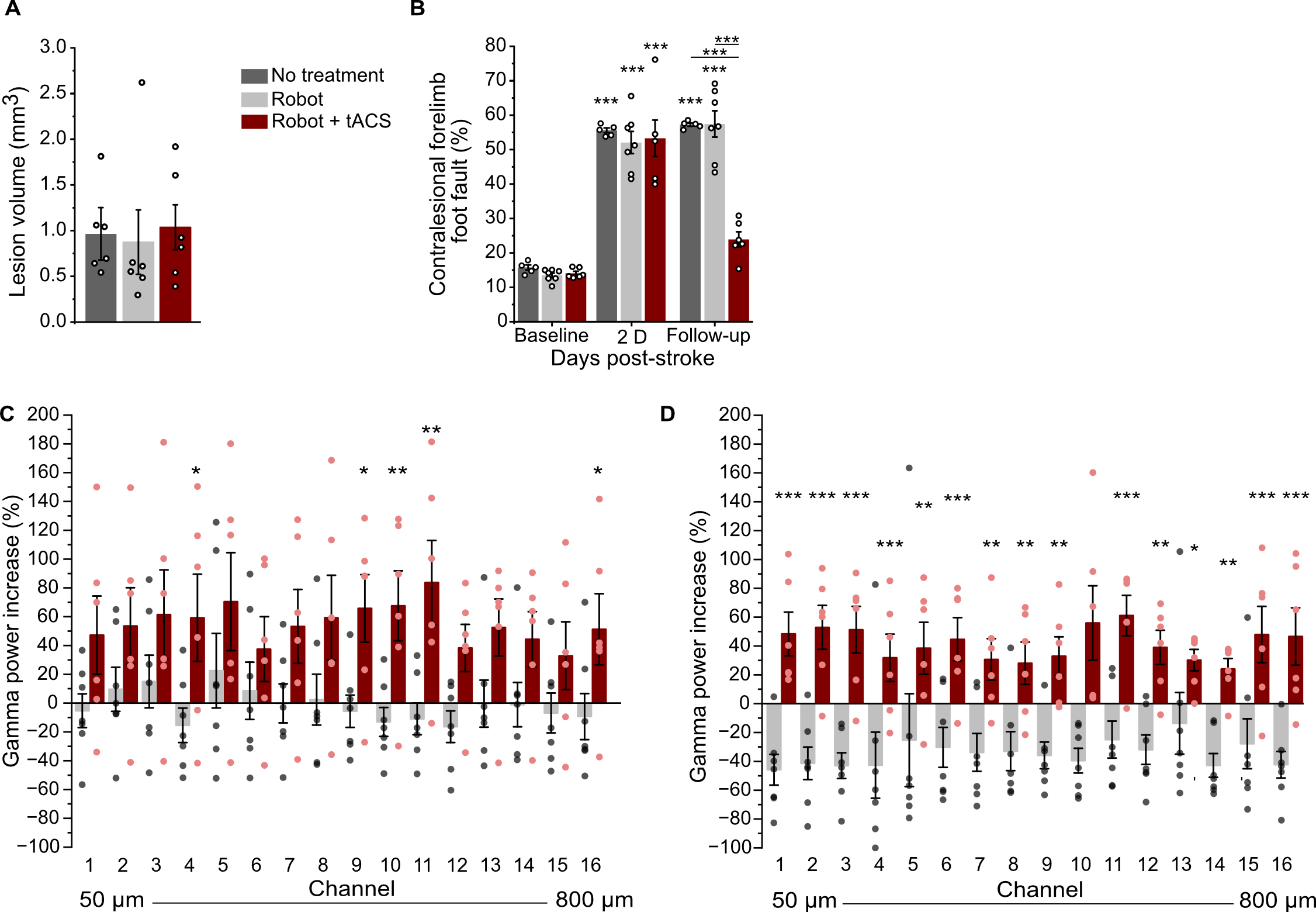

Supplement: S3 Fig — A, Quantification of lesion volume in stroke-affected animals that underwent no intervention (No treatment, dark gray, n = 6), robotic rehabilitation alone (Robot, light gray, n = 6) and robotic rehabilitation in combination with tACS (Robot + tACS, red, n = 6). No significant differences were observed in the lesion volume between the three groups. B, Motor performance assessment with the gridwalk test. Animals displayed a significant level of motor impairment at D2 postlesion. However, only the robot-tACS group showed performance comparable to baseline during the follow-up period (FU). (Two-way RM ANOVA followed by Tukey test, * = P < 0.05, ** = P < 0.01, *** = P < 0.001.) C and D, Quantification of gamma band power across all the 16 channels spanning all the cortical layers (channel 1 ≃ 50 μm, channel 16 ≃ 800 μm) in pre- and postonset windows, respectively. Mice treated with rehabilitation alone (gray bar plots, n = 7) and coupled with noninvasive 40 Hz tACS (red bar plots, n = 6). Two-way ANOVA followed by Tukey test, * P < 0.05, *** P < 0.001. Each dot represents a single animal. Data are shown as mean ± SEM. The data underlying this figure can be found in https://data.mendeley.com/datasets/mw82tzp4rx/1. (TIFF) [file pbio.3002806.s003.tiff]

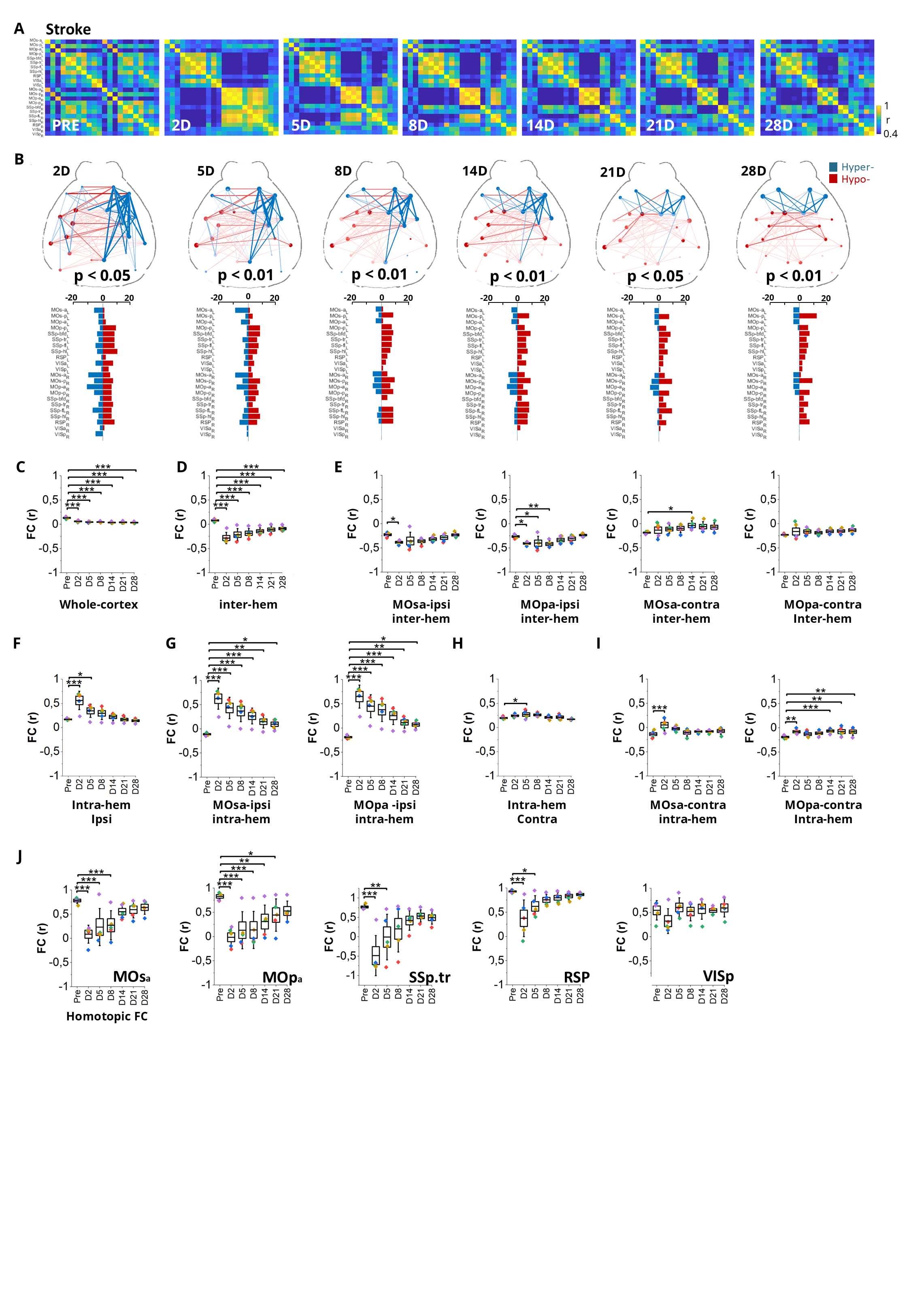

Supplement: S4 Fig — A, Pairwise Pearson’s correlation coefficients of cortical activity for each imaging time point in the stroke group after global signal regression. B, Network diagrams of statistically significant FC alterations after 2, 5, 8, 14, 21, or 28 days from injury. Blue and red lines denote significant hyper-correlation and hypo-correlation compared to prestroke values, respectively. The bar plots (bottom) indicate the number of significant FC alterations for each cortical area. C, Box chart illustrating FC averaged over the whole cortex. D, Box chart illustrating the averaged inter-hemispheric FC. E, Box charts showing inter-hemispheric FC of ipsilesional and contralesional secondary and primary motor cortices in the anterior regions (MOsa and MOpa respectively). F, Intra-hemispheric FC of ipsi-lesional areas. G, Box charts displaying intra-hemispheric FC of the secondary (left) and primary (right) ipsilesional motor cortices in the anterior region. H, Intra-hemispheric FC of contralesional hemisphere. I, the box charts display intra-hemispheric FC of the secondary (left) and primary (right) contralesional motor cortices in the anterior region. J, Homotopic FC changes from prestroke to 28 days after injury (MOsa, anterior secondary motor cortex; MOsp, anterior primary motor cortex; SSp.tr, primary somatosensory cortex-trunk; RSP, dorsal part of the retrosplenial cortex; VISp, primary visual cortex). One-way ANOVA followed by Tukey test, * P < 0.05, ** P < 0.01, *** P < 0.001. Data are shown as mean ± SEM. Each color indicates a single subject, n = 5. The data underlying this figure can be found in https://data.mendeley.com/datasets/mw82tzp4rx/1. (TIF) [file pbio.3002806.s004.tif]

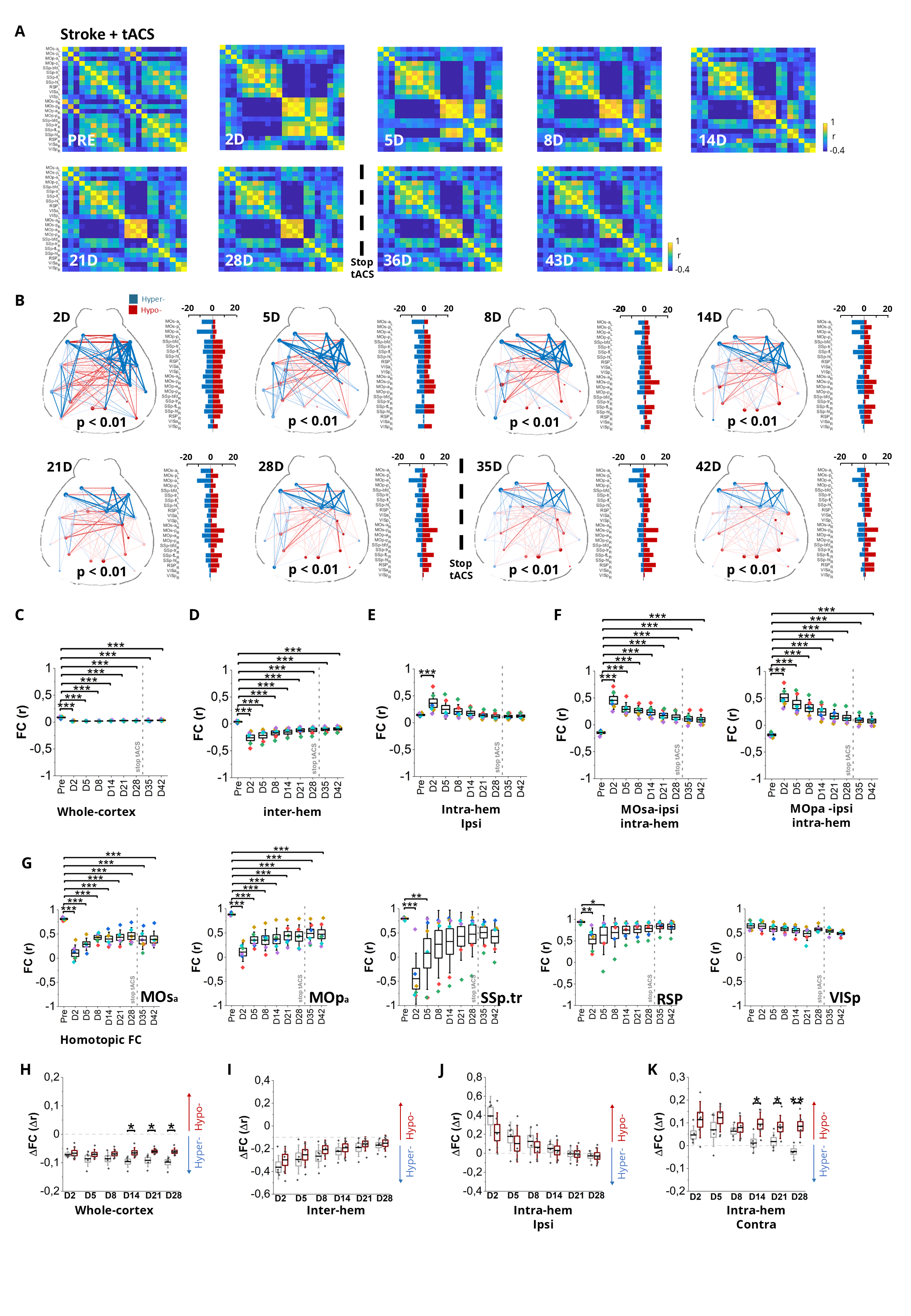

Supplement: S5 Fig — A, Pairwise Pearson’s correlation coefficients of cortical activity for each imaging time point in the Stroke + tACS group after global signal regression. B, Network diagrams of statistically significant FC alterations after 2, 5, 8, 14, 21, or 28 days from injury. Blue and red lines denote significant hyper-correlation and hypo-correlation compared to prestroke values, respectively. The bar plots (bottom) indicate the number of significant FC alterations for each cortical area. C, Box chart illustrating FC averaged over the whole cortex. D, Box chart illustrating the averaged inter-hemispheric FC. E, Intra-hemispheric FC of ipsi-lesional areas. F, Box charts displaying intra-hemispheric FC of the secondary (left) and primary (right) ipsilesional motor cortices in the anterior region. G, Homotopic FC changes from prestroke to 28 days after injury (MOsa, anterior secondary motor cortex; MOsp, anterior primary motor cortex; SSp.tr, primary somatosensory cortex-trunk; RSP, dorsal part of the retrosplenial cortex; VISp, primary visual cortex). One-way ANOVA followed by Tukey test, * P < 0.05, ** P < 0.01, *** P < 0.001. Data are shown as mean ± SEM. Each color indicates a single subject, n = 6. H-K Comparison of changes in functional connectivity (ΔFC) relative to baseline (prestroke) between Stroke (gray) and Robot + tACS (dark red) groups across different time points after stroke (D2–D28) in terms of whole-cortex (H), inter-hemispheric connectivity (I), intra-hemispheric connectivity of ipsilesional hemisphere (J), and intra-hemispheric connectivity of the contralesional hemisphere (K). Data are shown as box plots (mean ± SE). *p < 0.05, main effect of treatment (two sample t test). Blue and red arrows indicate the directions of hyper- and hypo-connectivity compared to the prestroke FC, respectively (stroke n = 5 mice, robot + tACS n = 6 mice). The data underlying this figure can be found in https://data.mendeley.com/datasets/mw82tzp4rx/1. (TIF) [file pbio.3002806.s005.tif]

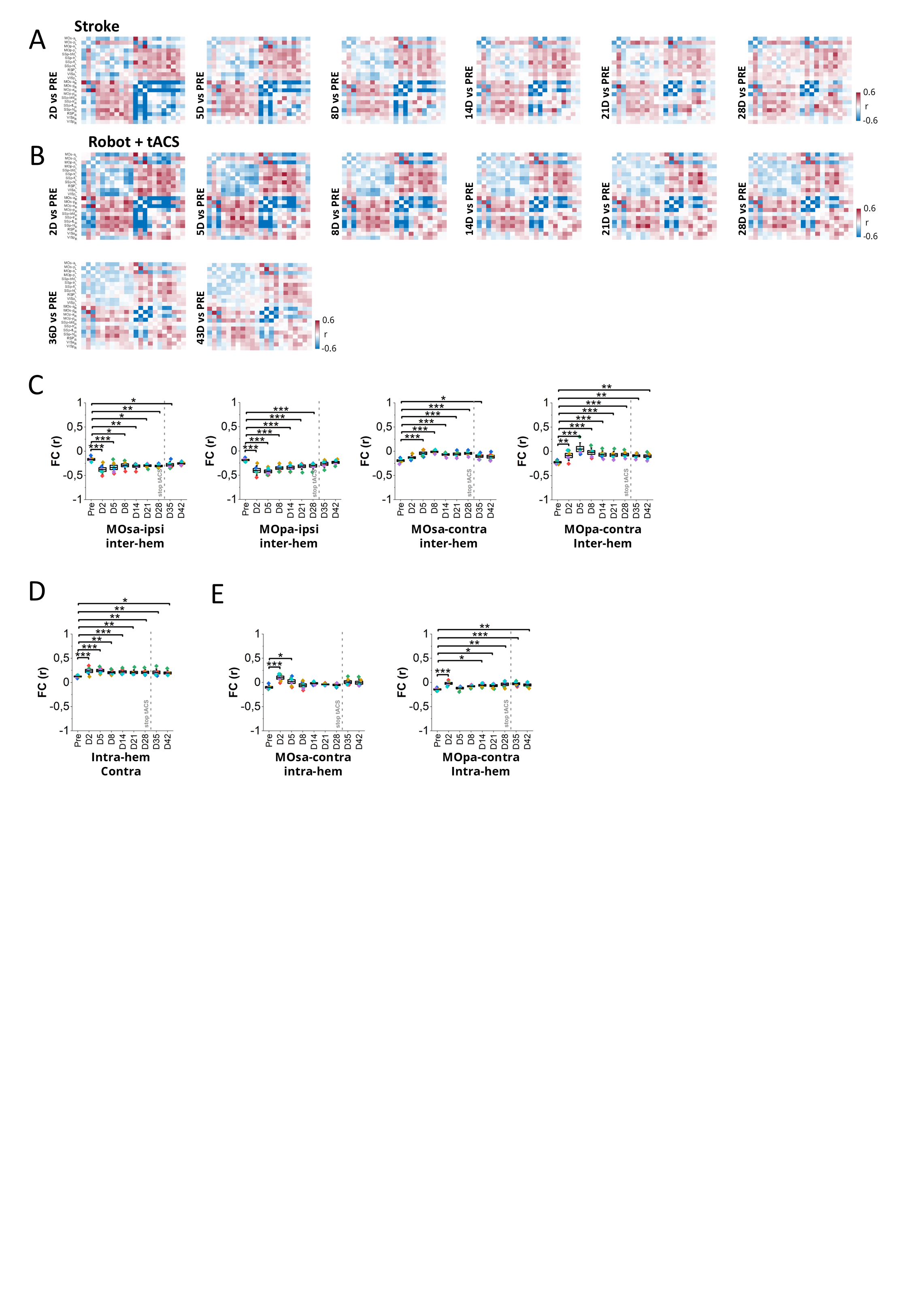

Supplement: S6 Fig — A, the averaged difference correlation matrices, produced by subtracting the poststroke FC (2, 5, 8, 14, or 30 days after injury) from prestroke FC in the stroke group after global signal regression. Red squares indicate poststroke hypo-connectivity, blue squares indicate poststroke hyper-connectivity of PV-IN. B, the averaged difference correlation matrices, produced by subtracting the poststroke FC (2, 5, 8, 14, or 30 days after injury) from prestroke FC in the Robot + tACS group after global signal regression. Red squares indicate poststroke hypo-connectivity, blue squares indicate poststroke hyper-connectivity of PV-IN. C, Box charts showing inter-hemispheric FC of ipsilesional and contralesional secondary and primary motor cortices in the anterior regions (MOsa and MOpa, respectively) for the Robot tACS group. D, Intra-hemispheric FC of contralesional areas for the Robot tACS group. E, The box charts displaying intra-hemispheric FC of the secondary (left) and primary (right) contralesional motor cortices in the anterior region for the Robot tACS group. One-way ANOVA followed by Tukey test, * P < 0.05, ** P < 0.01, *** P < 0.001. Data are shown as mean ± SEM. Each color indicates a single subject, n = 6. The data underlying this figure can be found in https://data.mendeley.com/datasets/mw82tzp4rx/1. (TIF) [file pbio.3002806.s006.tif]

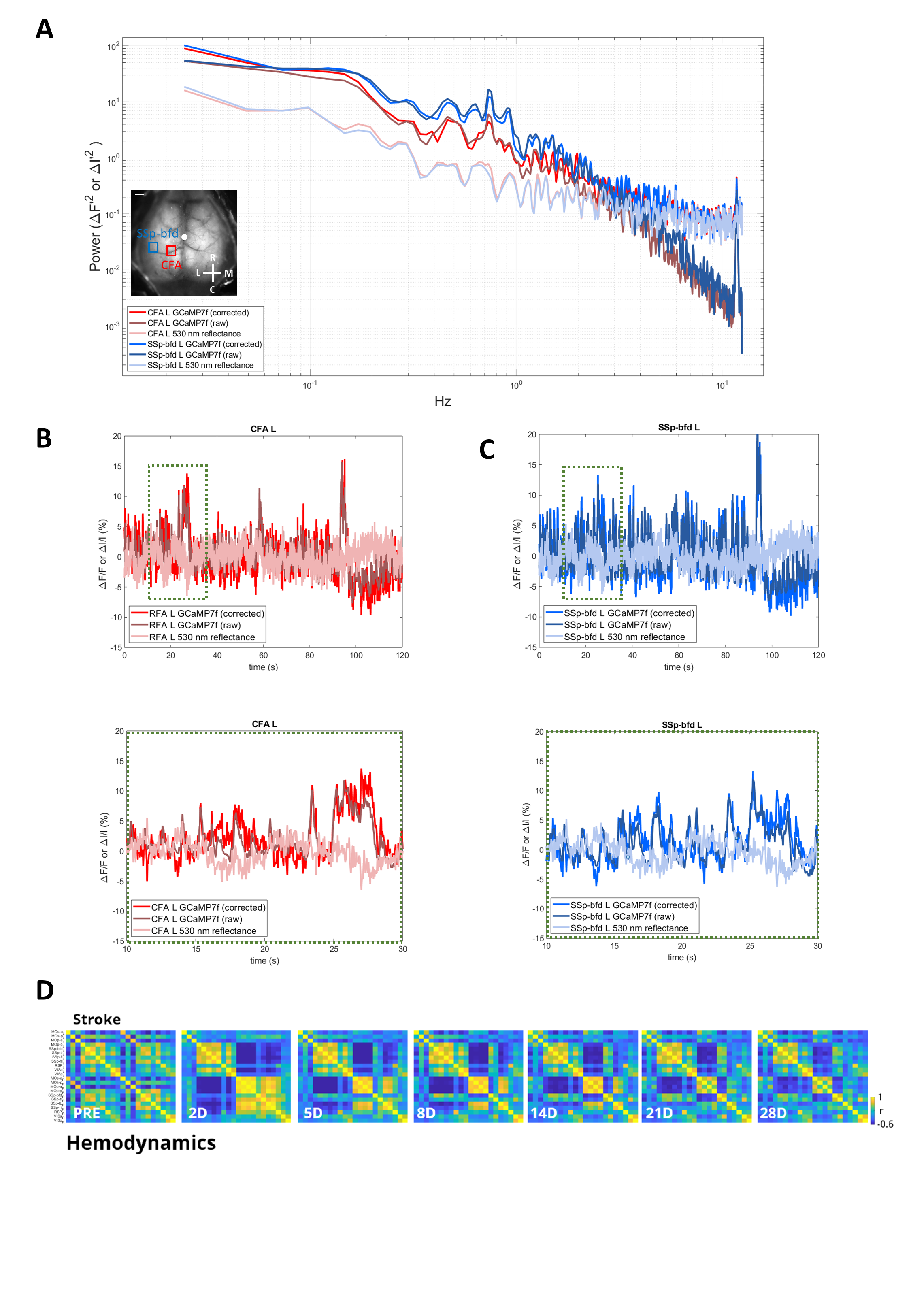

Supplement: S7 Fig — A, Power spectral density (PSD) of GCaMP7f fluorescence and 530 nm reflectance signals from left CFA region (in red) and left SSp-bfd region (in blue) in wake resting-state condition prestroke (ROI: 5 × 5 pixels, FOV: 128 × 128 pixels). These regions are clearly indicated in the inset of the figure (scale bar: 1 mm, white dot indicates bregma). For each region, we report the raw (uncorrected) GCaMP7f signal (darker color), the signal after correction for hemodynamic contamination (palet tone), and the corresponding 530 nm reflectance trace (lighter color). Data are plotted on a log–log scale to highlight frequency-dependent power and the presence of 1/f dynamics (n = 1). B, The upper plot shows representative time courses of raw GCaMP7f fluorescence (ΔF/F, dark red) and 530 nm reflectance (ΔI/I, light red) signals extracted from the left CFA region (CFA L) during a 120-s widefield optical imaging (WFOI) session. Corrected GCaMP7f fluorescence (bright red) was obtained by regressing out the 530 nm reflectance signal from the raw GCaMP7f signal, to reduce contamination from hemoglobin absorption. The bottom panel presents a magnified view of the time window highlighted in green in the upper plot (10–30 s), allowing clearer visualization of the temporal relationships between the raw and corrected GCaMP7f signals and the hemodynamic component. C, The upper plot shows representative time courses of raw GCaMP7f fluorescence (ΔF/F, dark red) and 530 nm reflectance (ΔI/I, light red) signals extracted from the left barrelfield region (SSp-bfd L) during a 120-s widefield optical imaging (WFOI) session. Corrected GCaMP7f fluorescence (bright red) was obtained by regressing out the 530 nm reflectance signal from the raw GCaMP7f signal, to reduce contamination from hemoglobin absorption. The bottom panel presents a magnified view of the time window highlighted in green in the upper plot (approximately 10–30 s), allowing clearer visualization of the temporal relationships between [file pbio.3002806.s007.tif]

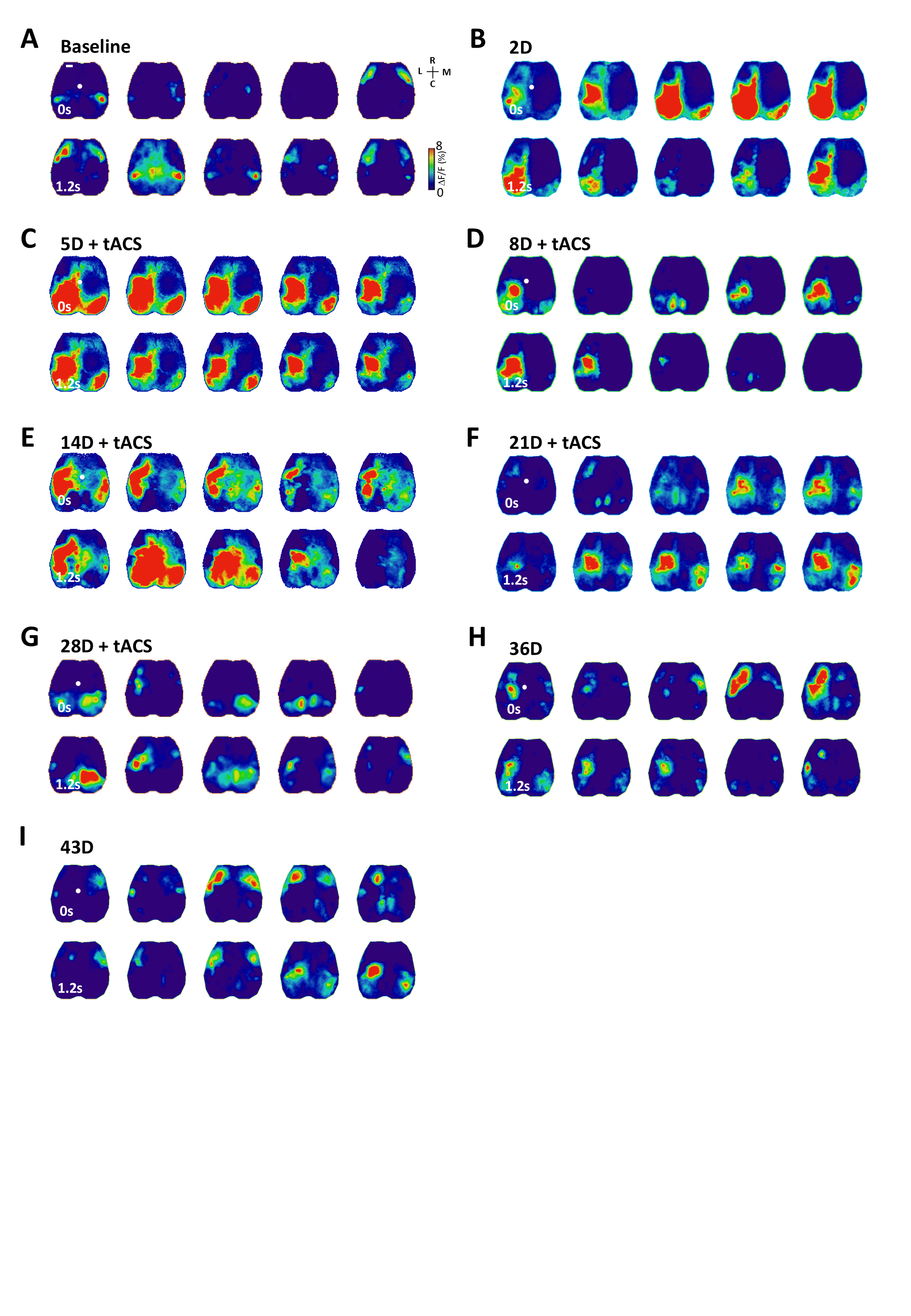

Supplement: S8 Fig — Representative PV-Cre::GCaMP7f resting state cortical activity maps in a mouse at baseline (A) and after 2 (B), 5 (C), 8 (D), 14 (E), 21 (F), 28 (G), 36 (H), and 43 (I) days after stroke. From day 5 to day 28, the mouse received daily tACS. L: lateral; M: medial; R: rostral; C: caudal. Scale bar: 1 mm. The data underlying this figure can be found in https://data.mendeley.com/datasets/mw82tzp4rx/1. (TIF) [file pbio.3002806.s008.tif]
